# Prevalence of depression in Uganda: A systematic review and meta-analysis

**DOI:** 10.1371/journal.pone.0276552

**Published:** 2022-10-20

**Authors:** Mark Mohan Kaggwa, Sarah Maria Najjuka, Felix Bongomin, Mohammed A. Mamun, Mark D. Griffiths

**Affiliations:** 1 Department of Psychiatry, Mbarara University of Science & Technology, Mbarara, Uganda; 2 African Centre for Suicide Prevention and Research, Mbarara, Uganda; 3 Department of Psychiatry and Behavioural Neurosciences, Forensic Psychiatry Program, McMaster University, Hamilton, Ontario, Canada; 4 School of Medicine, College of Health Sciences, Makerere University, Kampala, Uganda; 5 Department of Medical Microbiology & Immunology, Faculty of Medicine, Gulu University, Gulu, Uganda; 6 CHINTA Research Bangladesh, Savar, Dhaka, Bangladesh; 7 Department of Public Health and Informatics, Jahangirnagar University, Savar, Dhaka, Bangladesh; 8 Psychology Department, Nottingham Trent University, Nottingham, United Kingdom; University of Tripoli, LIBYA

## Abstract

**Background:**

Depression is one of the most studied mental health disorders, with varying prevalence rates reported across study populations in Uganda. A systematic review and meta-analysis was carried out to determine the pooled prevalence of depression and the prevalence of depression across different study populations in the country.

**Methods:**

Papers for the review were retrieved from *PubMed*, *Scopus*, *PsycINFO*, *African Journal OnLine*, and *Google Scholar* databases. All included papers were observational studies regarding depression prevalence in Uganda, published before September 2021. The Joanna Briggs Institute Checklist for Prevalence Studies was used to evaluate the risk of bias and quality of the included papers, and depression pooled prevalence was determined using a random-effects meta-analysis.

**Results:**

A total of 127 studies comprising 123,859 individuals were identified. Most studies were conducted among individuals living with HIV (*n* = 43; 33.9%), and the most frequently used instrument for assessing depression was the Depression sub-section of the Hopkins Symptom Checklist (*n* = 34). The pooled prevalence of depression was 30.2% (95% confidence interval [CI]: 26.7–34.1, *I*^*2*^ = 99.80, *p*<0.001). The prevalence of depression was higher during the COVID-19 pandemic than during the pre-pandemic period (48.1% vs. 29.3%, *p* = 0.021). Refugees had the highest prevalence of depression (67.6%; eight studies), followed by war victims (36.0%; 12 studies), individuals living with HIV (28.2%; 43 studies), postpartum or pregnant mothers (26.9%; seven studies), university students (26.9%; four studies), children and adolescents (23.6%; 10 studies), and caregivers of patients (18.5%; six studies).

**Limitation:**

Significantly high levels of heterogeneity among the studies included.

**Conclusion:**

Almost one in three individuals in Uganda has depression, with the refugee population being disproportionately affected. Targeted models for depression screening and management across various populations across the country are recommended.

**Trial registration:**

Protocol registered with PROSPERO (CRD42022310122).

## Introduction

The global prevalence rates of depression and other mental disorders have been increasing since 1990 [1], and approximately 3.8% of individuals worldwide have depression [2]. The prevalence of depression increased by an additional 27.6% globally during the coronavirus disease 2019 (COVID-19) pandemic (2020 to 2021) and by 23% in sub-Saharan Africa [3]. Despite the lower increase in the burden of depression in sub-Saharan Africa, most individuals with depression go undiagnosed and untreated in the region [4]. This has led to some individuals having extreme complications of depression, such as suicide [5–11]. The effect is even worse in low-income countries such as Uganda, with a high prevalence of depression (i.e., approximately one-fifth of the population between 2010 and 2017, and 27% of outpatients being depressed based on previous systematic review and meta-analyses [12, 13]).

In Uganda, due to the high burden of depression, various studies have been conducted among different populations (e.g., infected with human immunodeficiency virus [HIV], women, cancer patients, caregivers of patients, students, etc.) to understand its effects and design possible interventions [14–17]. Ugandan clinicians and researchers have employed various psychometrically validated tools to screen and diagnose depression among Ugandans, including the Patient Health Questionnaire (PHQ), Beck’s Depression Inventory (BDI), Hamilton Rating Scale for Depression, Symptom Checklist-20, Center for Epidemiologic Studies—Depression Scale, Akena Visual Depression Inventory, and the Mini-International Neuropsychiatric Interview (MINI) [18–24].

Uganda is a landlocked low-income country that has been affected by multiple adverse events, including civil wars, extreme poverty, high rates of HIV, various epidemics (e.g., Ebola), and poor mental health services, as well as being one of the largest refugee-hosting countries in the world [25–30]. These adverse events put many Ugandans at risk of developing depression due to the multiple physical, psychological, emotional, and social difficulties they are associated with. The effects of these difficulties are evidenced by the high levels of depression within various study groups in the country, such as women, children, students, individuals living with HIV, refugees, and members of the general public, with many groups reporting depression prevalence rates of over 70% [14, 31–37].

Due to the high burden of depression and a large amount of literature concerning mental health in Uganda, various systematic reviews have been conducted, especially among individuals living with HIV [12, 15, 38]. However, a comprehensive systematic synthesis of all published literature on depression in Uganda is lacking. Therefore, the present systematic review and meta-analysis aimed to determine the pooled prevalence of depression in Uganda and determine the prevalence of depression among various study populations in the country.

## Methods

This review was conducted in accordance with the Preferred Reporting Items for Systematic Reviews and Meta-Analyses (PRISMA) guidelines [39] and the Meta-analysis of Observational Studies in Epidemiology (MOOSE) guidelines for systematic reviews and meta-analysis of observational studies [40]. The study protocol was prospectively registered with PROSPERO (CRD42022310122). The review question was formed according to the Joanna Briggs Institute (JBI) Checklist for Prevalence Studies and the CoCoPop (Condition, Context, and Population) [41]. The *condition* was depression, the *context* was Uganda, and the *population* was all studied groups. Therefore, the review’s research questions were: (i) “What is the prevalence of depression in Uganda?” and (ii) “What is the prevalence of depression among various study populations in Uganda?”

### Search strategy

**Fig 1:**
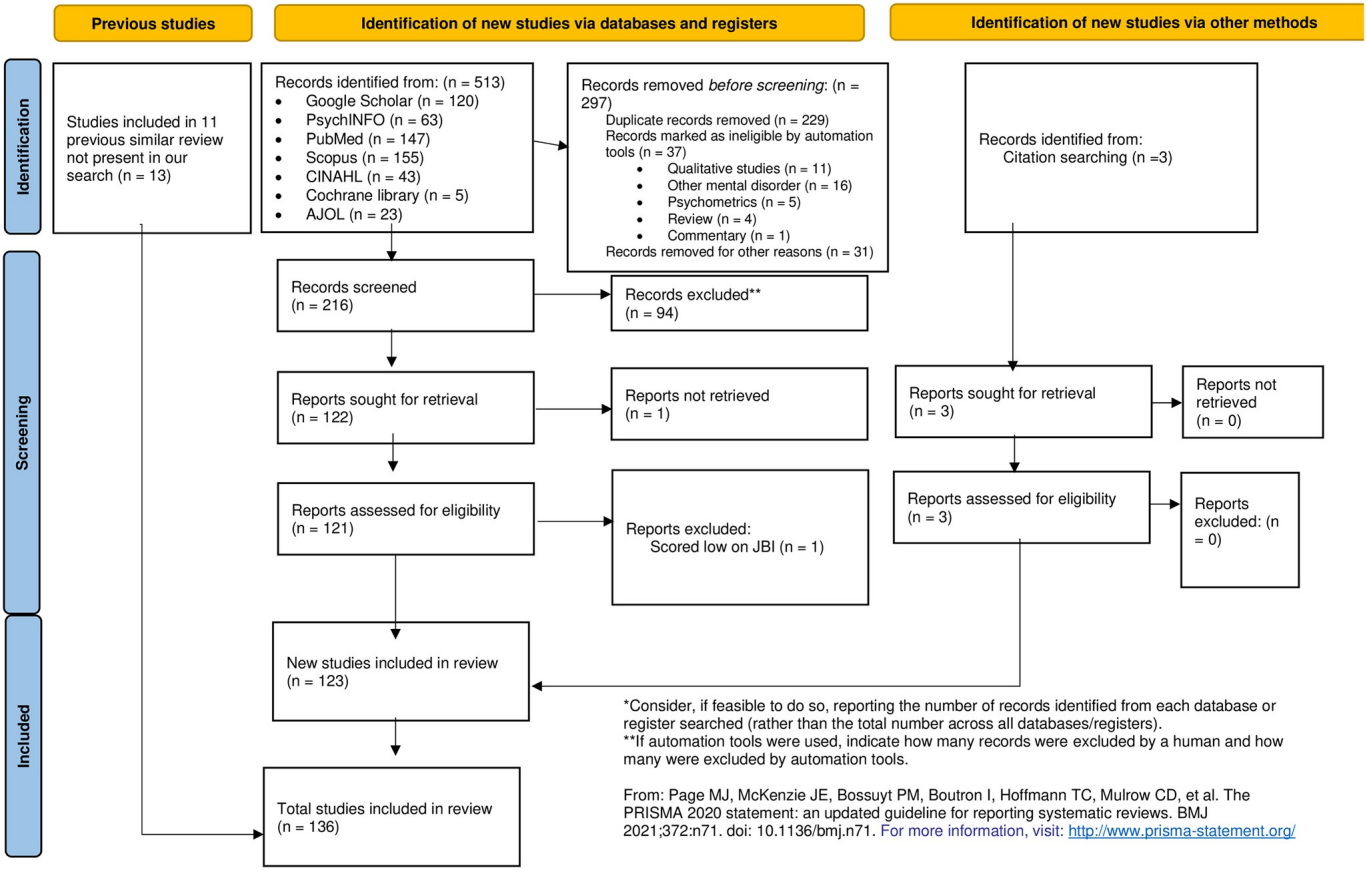
The PRISMA flow chart.

With the help of the university Librarian at Mbarara University of Science and Technology, relevant databases were used for the literature search, including *PubMed*, *Scopus*, *PsychInfo*, and *Google Scholar* (in the present review, eligible papers from the first 100 pages based on relevance were included), and *African Journal OnLine* (*AJOL*). The study included articles (both peer-reviewed and preprints) in all languages from 1972 (the first published paper about depression in Uganda [42]) to September 2021. The following key words were used in the literature search from different databases: (i) ‘depression,’ (ii) ‘Uganda,’ and (iv) ‘prevalence’ or ‘incidence.’ All systematic reviews concerning depression in Uganda, East Africa, and Africa were reviewed for eligible studies. The PRISMA 2020 flow chart shows the details of the search hits retrieved, included, or excluded papers [43] (Fig 1).

### Inclusion criteria and exclusion criteria

The literature search included all observational studies (cross-sectional, case-control, and cohort studies) published in all languages regarding depression prevalence (based on different assessment tools or subjective reports) in Uganda, based on various assessment tools and cut-offs; and excluded case reports, case series, qualitative research, letters to the editor, commentaries, conference proceedings or abstracts, policy papers, protocols, reviews, and meta-analyses.

### Study and data management

All identified papers were entered into *EndNote 9* to ascertain duplicates. After removing duplicates and review articles, the titles and abstracts of the articles were screened for inclusion independently by two team members. MAM settled any discrepancies after a discussion with the two team members for the reason of exclusion. The final selected papers were read for the full review, and this was done in pairs after dividing the results into two. Two members of the research team reviewed the first half, and another two members of the research team reviewed the second half. For papers whose full texts were not fully accessible, the corresponding authors were contacted by email. The papers were assessed for a quality check using the JBI Checklist for Prevalence Studies [44], as used in other systematic reviews [45]. Finally, a research team member screened for eligible papers from the systematic reviews regarding depression in the region.

### Data extraction

A pre-piloted and self-designed *Google Forms* document with the following information was used to collate the data. The data extracted included: the first author, year of data collection, study design, paper quality assessment questions based on the JBI Checklist, study group, sample size, age of participants, number of male and female participants, tools/questions used to assess for depression, and prevalence of depression.

### Bias evaluation and quality assessment of the included papers

The nine-item JBI Checklist was used to evaluate the risk of bias and the quality of the included papers [44]. The JBI Checklist uses a four-point response system: “no,” “yes,” “unclear,” and “not applicable” for the following study characteristics: (i) appropriateness of the sample frame; (ii) recruitment procedure; (iii) adequacy of the sample size; (iv) description of participants and setting; (v) description of the identified sample; (vi) validity of the methods used to screen for depression; (vii) reliability of the methods used to screen for depression; (viii) adequacy of statistical analyses; and (ix) response rate. Articles were assigned one point for each ‘*yes*’ response, and the remaining responses were assigned zero points. Therefore, the total score ranged from 0 to 9. Studies with a score of 4 or above were considered good quality. One article was excluded due to scoring poorly on most parameters [42]. The scores of the papers are presented in Table 1.

**Table 1:** Participants’ characteristics of all studies included in the meta-analysis.

First author, year of publication	Study design (JBI Checklist score)	Year of data collection	Districts	Study group	Sample size	Female (male)	Age (in years)	Tools used to access depression (cutoff)	Depression n (%)
Bolton 2004 [55]	CS (9)	2000	Masaka and Rakai	General population	587	364 (223)	39.3±2.9	Depression section of the Hopkins Symptom Checklist (DHSCL)	123 (21)
Ovuga 2006 [56]	CS (7)	2000 and 2002	Kampala	Non-medical undergraduate students in 2000 and undergraduate medical students in 2002	253 and 101	92 (161) and 31 (70)	21.3± 2.4 and 23.5± 5.0	Beck Depression Inventory (BDI)–(10)	37 (16.2) and 4 (4)
Nakasujja 2007 [57]	CS (9)	2001	Kampala	Elderly patients in non-psychiatric wards	127	64 (63)		Self-Reporting Questionnaire (SRQ 25)–(>5)	23 (18)
Nalugya-Sserunjogi 2016 [58]	CS (9)	2003	Mukono	school-going adolescents	519	218 (301)	16±2.18	Children Depression Inventory (CDI)–(19) MINI-KID	109 (21) and 8 (2.9)
Nakku 2006 [59]	CS (9)	2002–2003	Kampala	Women 6 weeks postpartum	523	523	23.4±4.76	MINI and SRQ-25 –(>5)	32 (6.1) and 38 (7.3)
Kaharuza 2006 [60]	RCT (9)	2003–2004	Bugiri, Busia, Mbale, and Tororo	Individuals living with HIV	1017	781 (236)	31–40 = 452; 31–40 = 452; 41–50 = 291; and 50+ = 94	Center for Epidemiological Studies Depression scale (CES-D)	476 (47)
Kinyanda 2011b [61]	CS (9)	2003–2004	Adjumani, Apac, Arua, Bugiri, Bushenyi, Kaberamaido, Kapchorwa, Katakwi, Lira, Moyo, Mubende, Nebbi, Soroti, and Yumbe	General populations	4,660			DHSCL–(>1.75)	1366 (29.3)
Ovuga 2005 [62]	CS (9)		Adjumani, Bugiri	General populations	524			BDI	91 (17.4)
Lundberg 2011 [63]	CS (9)	2004–2005	Kampala, Mbarara	Residents from urban and semi-urban Kampala and Mbarara	630	312 (334)	18–24 = 337; 25–30 = 437	DHSCL–(>1.75)	96 (9.84)
Nakimuli-Mpungu 2013a [64]	CS (8)	2004–2005	Kiruhura	Individuals living with HIV	244	156 (88)	36.2±8.9; Range = 18–60	DSMIV	97 (40)
Agardh 2012 [65]	CS (8)	2005	Mbarara	Undergraduate university students at MUST	976	362 (614)	Median age = 23. Younger ≤23: 628. Older>23: 329 (34.4), missing 23	DHSCL-25—(31)	146 (15)
Vinck 2007 [66]	CS (9)	2005	Gulu, Kitgum, Lira, and Soroti	War victims in displacement camps	2585	1293 (1292)	36.7±13.8	DHSCL–(40)	1151 (44.5)
Pfeiffer 2011 [67]	CS (7)	2005	Gulu	War victims	72	31 (41)	Median = 23.7	DHSCL–(>1.75)	51 (71)
Martinez 2008 [68]	CS (9)	2005	Mbarara	Individuals living with HIV	421	266 (155)	Median = 36, IQR = 11	DHSCL–(>1.75)	79 (18.8)
Muhwezi 2007 [69]	CS (9)		Kampala, Mpigi, and Mubende	Individuals attending primary health care facilities	199	119 (80)		MINI	74 (31.6)
Petrushkin 2005 [70]	CS (7)		Kampala	Individuals living with HIV	46	24 (22)	36.6	MINI	25 (54.35)
Psaros 2015 [71]	Cohort (9)	2005–2010	Mbarara	Uganda AIDS Rural Treatment Outcomes (UARTO) cohort study	453	314 (139)	34.9±8.3	DHSCL–(>1.75)	172 (37.97)
Nakasujja 2010 [72]	CS (8)	2005–2007	Kampala	HIV-positive patients are at risk for cognitive impairment, and HIV-negative patients	127	84 (43)		CES-D	62 (48.82)
Kaida 2014 [73]	Cohort (9)	2005–2012	Mbarara	Pregnant and postpartum HIV-positive women	447	447 (0)	Median = 32; IQR = 10	DHSCL–(>1.75)	173 (38.9)
Roberts 2008 [74]	CS (9)	2006	Amuru, Gulu	War victims	1210	727 (483)	35.4	DHSCL–(>1.75)	811 (67)
Klasen 2013 [75]	CS (9)	2006	Gulu	Child soldiers (children and adolescents of war abducted victims who became soldiers)	330	160 (170)	14.44±1.57	MINI-KID	120 (36.4)
Klasen 2010a [76]	CS (9)	2006	Gulu	Child soldiers (children and adolescents of war abducted victims who became soldiers)	330	160 (170)	14.44±1.57	MINI-KID	120 (36.4)
Klasen 2010b [77]	CS (9)	2006	Gulu	Child soldiers (children and adolescents of war abducted victims who became soldiers)	330	160 (170)	14.44±1.57	MINI-KID	120 (36.4)
Abbo 2009 [78]	CS (9)	2007	Iganga and Jinja	Clients of traditional healers	387	208 (178)	34.8±13.55	MINI	21 (5.4)
Hatcher 2012 [79]	Secondary data analysis (9)	2007	Mbarara	HIV-infected women	270	270 (0)	Median = 34. IQR = 10	DHSCL–(>1.75)	64 (6.7)
Pham 2009 [80]	CS (9)	2007	Amuria, Amuru, Gulu, Kitgum, Lira, Oyam, and Pader	War victims	2875	1417 (1458)	35.4±14.35	DHSCL–(42)	1150 (40)
Tsai 2012 [81]	Cohort (9)	2007–2010	Mbarara	Individuals living with HIV	456	324 (132)		DHSCL–(>1.75)	71 (15.57)
Tsai 2016 [82]	Cohort (9)	2007–2011	Mbarara	Individuals living with HIV	173	173 (0)	Median = 32; IQR = 11	DHSCL–(>1.75)	57 (33)
Nakimuli-Mpungu 2013b [83]	A prospective study (9)	2007–2012	Gulu, Kitgum, Soroti, Tororo	War victim with post-trauma disorder	2868			SRQ-20	1297 (45.22)
Winkler 2015 [84]	CS (9)	2008	Gulu, Kitgum, and Lira	child soldiers and war-affected victims	843	355 (488)	19.0±2.7.	DHSCL–(>2.65)	64 (7.6)
Musisi 2009 [85]	CS (8)		Kampala	HIV positive adolescents	82	46 (36)	13.4±1.8	SRQ-25	34 (40.8)
Nsereko 2018 [51]–Thesis	CS (9)	2018	Kampala	School adolescents	549	317 (228)		The Youth Self-Report (YSR)	115 (21.1)
Wagner 2012a [86]	Cohort (9)	2008–2009	Jinja, Kampala	Individuals living with HIV	602	409 (193)	35.7	Patient Health Questionnaire– 9 (PHQ-9)–(10)	78 (13)
Shumba 2013 [87]	CS (7)	2008–2009		Individuals living with HIV	732	504 (228)	19–39 = 294; 40–50 = 290; 50+ = 91	Developed own tool	432 (59)
Okeke 2013 [88]	Cohort (9)	2008–2010	Kampala	Individuals living with HIV	482		34.60±8.51	PHQ-9 –(10)	40 (8.3)
Wagner 2014a [89]	Cohort (9)	2008–2011	Jinja, Kampala, Mityana, and Mukono	3 study cohorts of Individuals living with HIV	750	435 (315)	34.5	PHQ-9 –(10)	45 (6)
Wagner 2017a [90]	Cohort (9)	2008–2011	Kampala, Mityana, Mukono, and Wakiso	Individuals living with HIV	1021	653 (368)	36	PHQ-9 –(10)	92 (9)
Wang 2018 [91]	CS (6)	2009	Wakiso	Individuals living with HIV	981			Self-report to a question, “*During the last 12 months*, *have you had a period lasting several days when you felt sad*, *empty*, *or depressed*?”	221 (22.5)
Ager 2012 [92]	CS (9)	2009	Amuru, Gulu	National Humanitarian Aid Workers	376	134 (238)	30.88±6.60	DHSCL–(>1.75)	256 (68)
Wagner 2011 [93]	CS (9)		Jinja, Kampala	New HIV clients attending the clinics	602	410 (192)	36; Range = 20–62	PHQ-9 –(10)	78 (13)
Ngo 2015 [34]	Cohort (9)	2009–2011	Mityana, Mukono, and Wakiso	Individuals living with HIV	1903	1492 (411)	36±9	PHQ-9–10 and MINI	1604 (84.3)
Kinyanda 2011a [94]	CS (9)	2010	Wakiso	Individuals living with HIV	618	449 (169)	18-24yrs = 58; 25–34 = 238; 35–44 = 217; and >44 = 103	MINI	50 (8.1)
Kinyanda 2012 [95]	CS (9)	2010	Wakiso	Individuals living with HIV	618	449 (169)	18-24yrs = 58; 25–34 = 238; 35–44 = 217; and >44 = 103	MINI	50 (8.1)
Morof 2014 [35]	CS (9)	2010	Kampala	female urban refugees	117	117 (0)	31.6±4.7	DHSCL–(>1.75) and DHSCL–(>2.65)	112 (92) and 70 (54)
Nakku 2013 [96]	CS (9)	2010	Wakiso	Individuals living with HIV	618	449 (169)	18-24yrs = 58; 25–34 = 238; 35–44 = 217; and >44 = 103	MINI	50 (8.1)
Kakyo 2012 [97]	CS (9)		Kabarole	Postpartum mothers	202	202 (0)	24±4.33	Edinburgh postpartum depression scale (EPDS)–(10)	87 (43)
Wagner 2012b [98]	Cohort (9)	2010–2011	Kampala, Mityana, and Mukono	Individuals living with HIV	798	530 (268)	36.1±9.5	MINI and PHQ-9 –(10)	111 (13.9) and 187 (.23.43)
Wagner 2014b [99]	Cohort (9)	2010–2011	Jinja, Kampala	Individuals living with HIV	1731	1131 (600)	36	PHQ-9 –(10)	156 (9)
Kiene 2018 [100]	Cohort (9)	2010–2011	Butambala	HIV positive and HIV negative clients	244	122 (122)	HIV positive = 34.60±9. 03; HIV negative = 33.28±10.12	DHSCL–(>1.75)	117 (48.0)
Okello 2015 [101]	Cohort (9)	2010–2011	Kampala	Individuals living with HIV	798	530 (268)	36.1± 9.53	PHQ-9 –(10)	100 (12.5)
Musisi 2014 [102]	Cohort (9)	2010–2011	Mukono, Wakiso, Kampala, and Mityana	Individuals living with HIV	386	225 (161)	35.7±8.7	PHQ-9 –(10)	116 (0.3)
Akena 2012 [103]	CS (9)	2011	Kampala	Individuals living with HIV	368	265 (103)	38.8±9.81; range = 18–71.	MINI	64 (17.4)
Akena 2013 [104]	CS (9)	2011	Kampala	Individuals living with HIV	735	525 (210)	38±10.08, range = 18–71	MINI	72 (9.8)
Nakimuli-Mpungu 2011a [105]	CS (9)	2011	Mubende	Individuals living with HIV	500	349 (151)	40±10.7, range: = 18–80	MINI	230 (46)
Katende 2017 [106]	CS (7)		Kampala	Caregivers of cancer patients	119	79 (40)	33±10.69	Hospital Anxiety and Depression Scale (HADS) standardized tool	
Kinyanda 2013 [107]	CS (9)		Gulu, Kaberamaido, Lira, and Tororo	Children and adolescent	1587	853 (734)	≤5 = 286; 6–9 = 416; 10–13 = 550; 14–19 = 335	MINI-KID	136 (8.6)
Spittal 2018 [108]	Cohort (9)	2011–2012	Amuru, Gulu, Nwoya	War victims—The Cango Lyec (Healing the Elephant) Project members 13 to 49 years who were sexually active	2008	1189 (819)	Range 13–49	DHSCL–(>1.75)	337 (16.78)
Whyte 2015 [109]	CS (5)	2011–2012	Agago, Amuru, Gulu, and Nwoya	OPD patients in several districts in northern Uganda	11325			Clinician diagnosis	40 (0.4)
Perkins 2018 [110]	CS (9)	2011–2012	Mbarara	General populations	1499	822 (677)	below 30 = 640	DHSCL–(>1.75)	268 (17.88)
Malamba 2016 [111]	Cohort (9)	2011–2012	Gulu, Nwoya, and Amuru	War victims—The Cango Lyec (Healing the Elephant) Project members 13 to 49 years	2388	1397 (991)	Range 13–49	DHSCL–(>1.75)	360 (14.9)
Hakim 2019 [112]	CS (9)	2011–2013	Kampala, Wakiso	All clients aged 13 or older attending Mildmay for client-initiated HIV testing and counseling	counseling	6998 (5234)	13–19 = 95; 20–24 = 2607; 25–34 = 5151; 35–49 = 3010; 50+ = 510	PHQ-2 –(4)	1884 (15.4)
Familiar 2019 [113]	Cohort (9)	2011–2014	Soroti	HIV-positive women attending a clinic	288	288 (0)	33.5; Range = 18–54	DHSCL–(>1.75)	139 (61)
Cavazos‑Rehg 2020 [114]	RCT (9)	2012–2017	Mbarara	HIV positive adolescents	592	335 (257)	12.13±0.65	CDI	298 (52.29)
Akena 2015 [115]	CS (9)	2013	Gulu, Kampala, and Mbarara	Patients with diabetes Mellitus	437	283 (154)	51±14.06, Range = 18–90	MINI	154 (34.8)
Familiar 2021 [116]	CS (9)	2013	Kampala	Self-settled Democratic Republic of Congo female refugees in Kampala	580	580 (0)	Mean = 33.7. 18–24 = 121; 25–34 = 210; 35+ = 249	PHQ-2	331 (57)
Akena 2016 [117]	Cohort (9)	2013	Luweero, Mityana, and Mpigi, Wakiso	Individuals living with HIV	1252	961 (291)	40±11. Range = 18–85	PHQ-9 –(10)	200 (67)
Rathod 2018 [118]	CS (9)	2013	Kamuli	General populations	1893	1500 (393)	Median = 28; IQR = 15	PHQ-9 –(10)	80 (4.2)
Mugisha 2016 [28]	CS (9)	2013	Amuru, Gulu, and Nwoya	Individuals in post-conflict northern Uganda	2361	1475 (886)	49% of respondents were aged above 34 years	MINI	599 (24.9)
Mugisha 2015 [29]	CS (9)	2013	Amuru, Gulu, and Nwoya	Community in the post-conflict northern Uganda	2361	1475 (886)	49% of respondents were aged above 34 years	MINI	599 (24.7)
Mwesiga 2015 [119]	CS (9)	2013	Kampala	Individuals living with HIV	345	245 (100)	Median = 35; (IQR = 12)	MINI	17 (5)
Nakku 2019 [120]	CS (9)	2013	Kamuli	General populations	1290	867 (423)	16–30 = 494; 31–49 = 555; ≥ 50 = 240	PHQ-9 –(10)	325 (25.4)
Nalwadda 2018 [121]	CS (9)	2013	Kamuli	The male population in the region	1129	0 (1129)	33% of participants were below 30 years	PHQ-9 –(10)	292 (25.9)
Jones 2017 [122]	Cohort (7)	2013	Kampala	Post-tuberculosis lung disease patients	29	14 (15)	45±13; Range = 17–69	PHQ-9 –(5)	7 (24)
Wagner 2017b [123]	Cohort (9)	2013	Luweero, Mityana, Mpigi, Wakiso	Individuals living with HIV	1252	976 (276)	39.8±11.2	PHQ-9 –(10)	375 (30)
Rukundo 2013 [124]	CS (9)		Mbarara	Physically ill patients	258	120 (138)	18–24 = 50, 25–40 = 130, 41–60 = 52	MINI	87 (33.7)
Olema 2014 [125]	CS (9)		Gulu	Adolescents and their parents in GULU	300			DHSCL–(>1.75)	120 (40)
Huang 2017 [126]	CS (9)	2013–2014		Parents of children in primary school	303	248 (55)	35.92±9.80; Range = 18–79	PHQ-9 –(10)	85 (28)
Wagner 2016a [127]	Cohort (9)	2013–2014	Luweero, Mityana, Mpigi, and Wakiso	Individuals living with HIV	1252	962 (290)	40±11.2	PHQ-9 –(10)	375 (30)
Wagner 2016b [128]	Cohort (9)	2013–2014	Luweero, Mityana, Mpigi, and Wakiso	Individuals living with HIV	1252	962 (290)	40±11.2	PHQ-9 –(10)	375 (30)
Henry 2019 [129]	CS (7)	2013–2015	Kampala	Adolescents attending an adolescent health clinic	514	372 (142)	16; Range = 10–19	Investigator designed tool (not standard)	174 (33.9)
Meffert 2019 [15]	Cohort (9)	2013–2017		Individuals living with HIV	475	282 (157)	18–33 = 146; 34–48 = 227; 49–63 = 69; 65+ = 5	CES-D	104 (22)
Swahn 2021 [130]	Cohort (9)	2014	Kampala	Individuals living with HIV—comparing HIV positive and non-HIV positive youth (12–18 years)	1096	614 (481)	12–14 = 219; 15–16 = 291; 17–18 = 586	Self-report—“*In the past year*, *did you ever feel so sad or hopeless almost every day for two weeks or more in a row that you stopped doing your usual activities*?”	673 (62)
Gyagenda 2015 [131]	CS (9)	2014	Kampala	Post-stroke patients	73	43 (30)	20–39 = 6; 40–59 = 25; 60–79 = 35; and 80–99 = 7	PHQ-9 –(10)	23 (31.5)
Fisher 2019 [31]	CS (6)	2014	Kisiro	Women attending OPD	115	115		SRQ-20	87 (75.65)
Kinyanda 2020 [132]	CS (9)	2014	Kampala, Masaka	caregivers of patients with HIV	1336			Child and Adolescent Symptom Inventory-5 (CASI-5)	Baseline = 66 (5.0); 6month = 47 (4.2); 12months = 43 (4.4)
Muhammad 2018 [133]	CS (6)	2014	Kabarole	Individuals living with HIV	150	84 (66)	16.53±5.24	-	37 (47)
Nakku 2016 [21]	CS (9)	2014	Kamuli	Patients attending a health facility	1415	1017 (398)	33±13	PHQ-9 –(8)	140 (10)
Ashaba 2015 [134]	Case-control (9)	2014	Mbarara	Mother of malnourished children	172	172 (0)	25±4.43; Range = 18–40	MINI	46 (26.7)
Kinyanda 2016a [135]	CS (9)		Amuria, Katakwi	Post-conflict communities	1110	631 (479)	56% were aged between 18 to 44 years.	DHSCL–(>1.75)	462 (41.6)
Manne-Goehler 2019 [136]	Cohort (9)		Mbarara	Older individuals (above 40 years) living with HIV and HIV uninfected individuals of similar sex	296	141 (155)	52	DHSCL–(>1.75)	81 (27.36)
Kinyanda 2016b [137]	CS (9)			Caregivers of patients with mental illness	468	292 (176)	All above 50	MINI	43 (9.19)
Smith 2019 [138]	CS (9)	2014–2015	Mbarara	General populations	1620	869 (751)		DHSCL–(>1.75)	460 (28.39)
Akimana 2019 [17]	CS (9)	2015	Kampala`	Children and adolescents with cancer	352	112 (240)	11.5±3.2; Range = 7–17	MINI-KID	91 (26)
Nampijja 2019 [139]	CS (9)	2015	Kampala	Postpartum women in Nsambya hospital	300	300 (0)	28±4.8; Range = 17–44	MINI	4 (1.3)
Cooper-Vince 2018 [140]	CS (9)	2015	Mbarara	Community-based study	1603	898 (705)		DHSCL–(>1.75)	461 (28.76)
Kiene 2017 [141]	CS (8)			Individuals living with HIV	325	165 (160)	Men = 34.93±10.59; Women = 32.21±8.93	DHSCL–(>1.75)	63 (19.4)
Kinyanda 2017b [142]	CS (9)		Masaka, Wakiso	Individuals living with HIV	899	705 (194)		MINI	126 (14.0)
Sohail 2019 [143]	CS (9)	2015–2017	Rakai	Rakai HIV community cohort	333	160 (170)	37± 9	CES-D	28 (8.4)
Raggio 2019 [144]	CS (8)	2016	Mbarara	Pregnant and non-pregnant women	225	225 (0)	Median = 27; IQR = 9	EPDS–(10) and DHSCL–(>1.75)	26 (11.56) and 60 (26.67)
Kinyanda 2017a [145]	Cohort (9)		Masaka, Wakiso	Individuals living with HIV	1099	847 (252)	35.1± 9.3	MINI	155 (14.1)
Akinbo 2016 [146]	CS (5)		Bushenyi	Alcoholic youth	204	71 (133)	30.6% were 15–19 years old	Researchers designed questionnaire	4 (2)
Cooper-Vince 2017 [147]	CS (9)		Mbarara	Female household head	257	257 (0)	33.5±7.9	DHSCL–(>1.75)	133 (44)
Ashaba 2021 [148]	CS (9)	2016–2017	Mbarara	HIV positive adolescents	224	131 (93)	14.8±1.4.	MINI-KID	37 (16)
Ashaba 2018 [149]	CS (9)	2016–2017	Mbarara	HIV positive adolescents	224	131 (93)	14.8±1.4.	MINI-KID	37 (16)
Ortblad 2020 [150]	Prospective studies (9)	2016–2019	Kampala	Individuals living with HIV	960		Median = 28, IQR = 8	PHQ-9	416 (43.2)
Satinsky 2021 [151]	CS (9)	2016–2018	Mbarara	General populations	1626	908 (718)	17–26 = 409; 27–39 = 488; 40+ = 705	DHSCL–(>1.75) and DSM V	331 (20.36) and 159 (9.78)
Alinaitwe 2021 [152]	CS (9)	2017	Kampala	TB patients	308	112 (188)	36±10.8	MINI	73 (23.7)
Bapolisi 2020 [153]	CS (9)	2017	Isingiro	Refugees in Nakivale refugee camp	387	219 (168)	33.01±12.2	MINI	224 (58)
Forry 2019 [154]	CS (9)	2017	Mbarara	Prison inmates in Mbarara Municipality	414	25 (389)	Aged 22–35 years (60%)	MINI	182 (44)
Seffren 2018 [155]	Cohort (9)		Busia, Tororo	caregivers of HIV patients	288	288 (0)	33.5±5.8	DHSCL–(>1.75)	28 (9.7)
Kuteesa 2020 [156]	CS (9)	2017	Mukono	15–24 years individuals in a fishing community	1281	606 (675)	Range = 15–24	PHQ-9 –(10)	29 (2)
Mubangizi 2020 [54]–Preprint	CS (9)	2017–2018	Kampala	Adults with sickle cell disease at Mulago Sickle cell clinic	255	161 (94)	Median = 21, IQR = 6	SRQ-25 –(>5)	174 (68.2)
Nabunya 2020 [157]	Longitudinal cluster randomized study (9)	2017–2022 (baseline)	Five districts in southwestern Uganda	Adolescent girls in southern Uganda	1260	1260 (0)	15.4±; Range = 14–15	BDI–(21)	580 (46.03)
Arach 2020 [158]	CS (9)	2018	Lira	Postpartum women	1789	1789 (0)	25±7; Range = 12–47	EPDS–(14)	377 (21.1)
Logie 2020 [159]	CS (9)	2018	Kampala	Refugee and displaced youth aged 16–24 living in five informal settlements in Kampala	445	333 (112)		PHQ-9 –(10)	297 (66.7)
Ssewanyana 2021 [32]	CS (7)	2018	Kampala	Patients with stomas	15	11 (3)	Range = 18–60	PHQ-9 –(5)	13 (88)
Musinguzi 2018 [160]	CS (9)		Masaka, Wakiso	Peri-urban Individuals living with HIV of Masaka and Entebbe	201	160 (41)	18–29 = 67; 30–34 = 39; 35–39 = 26; 40–49 = 48; 50+ = 21	MINI	62 (30.8)
Muliira 2019 [161]	CS (9)		Kampala	Caregivers of cancer patients	284	208 (76)	36±13.8	HADS–(8)	137 (48.2)
Lukenge 2019 [52]–Thesis	CS (9)	2018–2019	Kampala	Women attending infertility clinic	377	377 (0)		PHQ-9 –(10)	167 (44.3)
Misghinna 2020 [162]	CS (9)	2018–2019	Kampala	Refugees	374	218 (156)	37.1±12.1	MINI	174 (46.52)
Bahati 2021 [36]–Preprint	CS (5)	2019	Mbarara	Urban refugees in Mbarara municipality	343	145 (198)	28.8±11.0	PHQ-9 –(10)	329 (96)
Boduszek 2021 [163]	CS (9)	2019		Primary and secondary school students aged 9–17 years	11518	6035 (5483)	14±1.95	The 14-item Patient-Reported Outcomes Measurement Information System (PROMIS) Depression Short Form–(31)	1224 (10.62)
Kabunga 2020 [37]	CS (6)	2019	Isingiro	Refugees in Nakivale camp	146	76 (70)	18–30 = 26; 31–44 = 42; 44–59 = 45; and 60+ = 27	PHQ-9 –(10)	119 (81)
Kyohangirwe 2020 [164]	CS (9)	2019	Kampala	Adolescents	281	176 (105)	10–13 = 101; 14–17 = 180	MINI-KID	51 (18.2)
Olum 2020 [165]	CS (9)	2019	Kampala	Medical students at Makerere University	331	133 (196)	23.1±3.3	PHQ-9 –(10)	71 (21.5)
Mootz 2019 [166]	CS (9)		Amuria, Katakwi, and Kumi	Girls and women war victims	605	605 (0)		DHSCL–(30)	181 (30)
Ssebunnya 2019 [167]	CS (9)		Kamuli	General populations	1290	848 (442)	<25 = 281; 25–34 = 367; 35–44 = 302; >44 = 337	PHQ-9 –(10)	82 (6.4)
Kabunga 2021a [168]	CS (5)	2020	Isingiro	Refugees in Nakivale camp	146	77 (69)	Majority of respondents 31.5% (n = 46) were aged between 44 and 59	PHQ-9 –(10)	66 (45.2)
Bongomin 2021 [33]	CS (9)	2020	Kampala	Patients with rheumatoid arthritis	48	44 (4)	Median = 52; IQR = 17	EQ-5D-5L anxiety/depression dimension	34 (70.8)
Kabunga 2021b [169]	CS (9)	2020	Lira	Out-of-school adolescents	164	87 (77)	10–13 = 13; 14–16 = 48; 17–19 = 103	PHQ-9 –(10)	55 (34)
Kizito 2020 [53]–Thesis	Cohort (9)	2020	Masaka	Postpartum mothers	167	167 (0)	The majority were between 25–29	EPDS	58 (34.7)
Ouma 2021 [170]	CS (9)	2020	Gulu	Female sex workers	300	300 (0)	26.4±6.0	MINI	143 (47.7)
Najjuka 2020 [14]	CS (9)	2020		University students	321	123 (198)	24.8±5.1	Depression Anxiety and Stress Scale (DASS-21)	259 (80.7)
Mahmud 2021 [171]	Cohort (9)	2020	Kyenjojo	General population	1075			PHQ-9 –(10)	150 (14)
Nyundo 2020 [6]	CS (9)		Iganga, Mayuge	Adolescents (10–19)	598	286 (312)	14.2±2.6	the 6-Item Kutcher Adolescent Depression Scale (KADS-6)	158 (26.5)
Kaggwa 2021 [16]	CS (9)	2021	Isingiro	Married/cohabiting women	153	153 (0)	33.3±6.7.	PHQ-9 –(10)	100 (65.4)

Note: Table studies have been sorted according to the year of data collection. In those studies without the year of data collection, the year of submission to the journal was used to estimate the year of data collection. CS = Cross-sectional; DHSCL = Depression sub-section of the Hopkins Symptom Checklist; PHQ = Patient Health Questionnaire (PHQ); MINI = Mini-International Neuropsychiatric Interview; MINI-KID = Mini-International Neuropsychiatric Interview for Children and Adolescents; SRQ = Self-Reporting Questionnaire; CES-D = Center for Epidemiological Studies Depression Scale; EPDS = Edinburgh Postpartum Depression Scale; BDI = Beck Depression Inventory; CDI = Children Depression Inventory; HADS = Hospital Anxiety and Depression Scale; DSM = Diagnostic and Statistical Manual of Mental Disorders; CASI-5 = Child and Adolescent Symptom Inventory-5; DASS-21 = 21-item Depression Anxiety and Stress Scale; EQ-5D-5L = European Quality of Life Five Dimension-Five Level anxiety/depression dimension; KADS-6 = Kutcher Adolescent Depression Scale; PROMIS = Patient-Reported Outcomes Measurement Information System Depression Short Form

### Ethical considerations

The present study was a secondary analysis of previously published literature. Therefore, formal clearance by a Research and Ethics Committee was not required.

### Data synthesis and analysis

Data for this analysis are available at figshare [46]. *Microsoft Office 2016* (Microsoft Inc., Washington, USA) and *STATA 16*.*0 software* (Stata Corp LLC, College Station, Texas, USA) were used for data cleaning and statistical analysis. Descriptive statistics and qualitative narrative analysis were used to present individual study and participant characteristics. A random-effects meta-analysis [47] was performed using the *meta* command to determine the pooled prevalence of depression and prevalence of depression in the different study groups. The results were presented on forest plots. The Higgins Inconsistency index (*I*^2^) and univariate random effect meta-regression [48] were used to evaluate the heterogeneity among the selected studies. Publication bias was assessed visually using funnel plots symmetry [49], and fill and trim estimated the number of missing studies [50]. Egger’s test was also used to assess for small study effects. Univariate meta-regression was used to determine the source of heterogeneity based on the following: mean age, number per gender (males or females), data collection period (pre-COVID-19 pandemic vs. during the pandemic), study design, JBI Checklist score, sample size, and depression assessment tool used. Subgroup analysis was also conducted based on study types, study tools used, the diagnostic status of the tool, and the data collection period.

## Results

A total of 136 papers met the criteria for inclusion in the review (comprising three theses [51–53], two preprints [36, 54], and 131 peer-reviewed journal papers). Using *Microsoft Excel 2016*, duplicate papers were automatically identified [including republished datasets in different papers] (n = 9) based on year of data collection, type of study, district of study, sample size, study population, the prevalence of depression, and assessment tool used for depression. The remaining 127 papers, comprising a total of 123,859 individuals, comprised the total study sample (Table 1).

The identified papers were published between 2004 and 2021, and the data included were collected between 2000 and 2021 from 45 districts in Uganda.

Most of the studies were conducted in the capital city, Kampala (n = 43), followed by the districts of Mbarara (n = 23) and Gulu (n = 16). The pooled mean age of the participants was 27.19 years (95% CI: 24.31–30.09 years; *I*^2^ = 85.38, *p*<0.001). A total of eight studies were conducted during the COVID-19 pandemic [6, 14, 16, 33, 53, 168–171].

### Tools used in assessing depression

Both diagnostic and screening tools were used to assess depression among different populations in Uganda (Table 1). The tools used included: (i) Depression sub-section of the Hopkins Symptom Checklist (DHSCL) (*n* = 34, 26.8%) [35, 55, 61, 63, 65–68, 71, 73, 74, 79–82, 84, 92, 100, 108, 110, 111, 113, 125, 135, 136, 138, 140, 141, 144, 147, 151, 155, 166], (ii) Patient Health Questionnaire (PHQ) (*n* = 33, 26.0%) [16, 21, 32, 34, 36, 37, 52, 86, 88–90, 93, 98, 99, 101, 102, 112, 116–118, 120–123, 126–128, 131, 150, 156, 159, 165, 167–169, 171], (iii) Mini-International Neuropsychiatric Interview (MINI) (*n* = 24, 18.9%) [28, 29, 34, 59, 69, 70, 78, 94–96, 98, 103–105, 115, 119, 124, 134, 137, 139, 142, 145, 152–154, 160, 162, 170], (iv) Mini-International Neuropsychiatric Interview for Children and Adolescents (MINI-KID) (*n* = 6, 4.7%) [17, 58, 75–77, 107, 148, 149, 164], (v) Self-Reporting Questionnaire (SRQ) (*n* = 6, 4.7%) [31, 54, 57, 59, 83, 85] (vi) Center for Epidemiological Studies Depression Scale (CES-D) (n = 4, 3.1%) [15, 60, 72, 143], (vii) Edinburgh Postpartum Depression Scale (EPDS) (*n* = 4, 3.1%) [53, 97, 144, 158], (viii) Beck Depression Inventory (BDI) (*n* = 3, 2.4%) [56, 62, 157], (ix) Children Depression Inventory (CDI) (*n* = 2, 1.6%) [58, 114], (x) Hospital Anxiety and Depression Scale (HADS) standardized tool (*n* = 2, 1.6%) [106, 161], (xi) Diagnostic and Statistical Manual of Mental Disorders (DSM) (*n* = 2, 1.6%) [64, 151], (xii) Child and Adolescent Symptom Inventory-5 (CASI-5) (*n* = 1, 0.8%) [132], (xiii) European Quality of Life Five Dimension-Five Level (EQ-5D-5L) anxiety/depression dimension (*n* = 1, 0.8) [33], (xiv) the six-item Kutcher Adolescent Depression Scale (KADS-6) (*n* = 1, 0.8%) [6], (xv) the 14-item Patient-Reported Outcomes Measurement Information System (PROMIS) Depression Short Form (*n* = 1, 0.8%) [163], and (xvi) the Youth Self-Report (YSR) (*n* = 1) [51]. Two studies used a single self-report question to screen for depression [91, 130], and three studies developed their own tool to assess for depression [87, 129, 146]. One study used a clinician’s diagnosis of depression [109]. The DASS-21 was also used in one study [14]. Six studies used more than one tool to screen and/or diagnose depression [34, 58, 59, 98, 144, 151].

### Prevalence of depression

**Fig 2:**
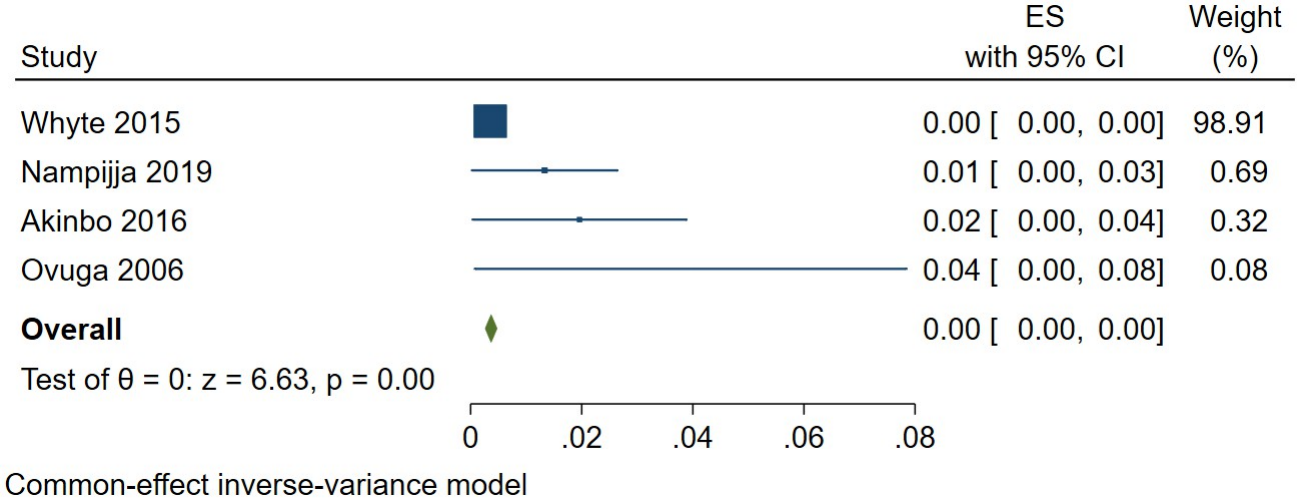
Forest plot showing the pooled prevalence of depression in Uganda following sensitivity analysis.

A total of 27,989 (out of 123,859) individuals screened positive for depression. The pooled prevalence of depression was 30.2% (95% confidence interval [CI]: 26.7%-34.1%; *I*^*2*^ = 99.80, *p*<0.001). The funnel plot was asymmetrical consistent with publication bias (S1 Fig). Despite the asymmetry, no studies were missing based on trim and fill analysis. The estimated slope from Egger’s test was 6.12 (standard error [*SE*] = 0.598, *p*<0.001), suggesting publication bias due to small study effects. A sensitivity analysis was performed using the four studies within the funnel [56, 109, 139, 146]. The pooled prevalence of depression was 0.9% (95% CI: 0.1%-1.7%; *I*^*2*^ = 37.82, *p* = 0.021) (Fig 2). In univariate meta-regression analysis (to explore potential sources of heterogeneity), depression increased with the use of DASS-21 or SRQ-20 assessment tools and studies conducted during the COVID-19 pandemic. However, depression decreased with increased sample size and the number of females in the study.

**Fig 3:**
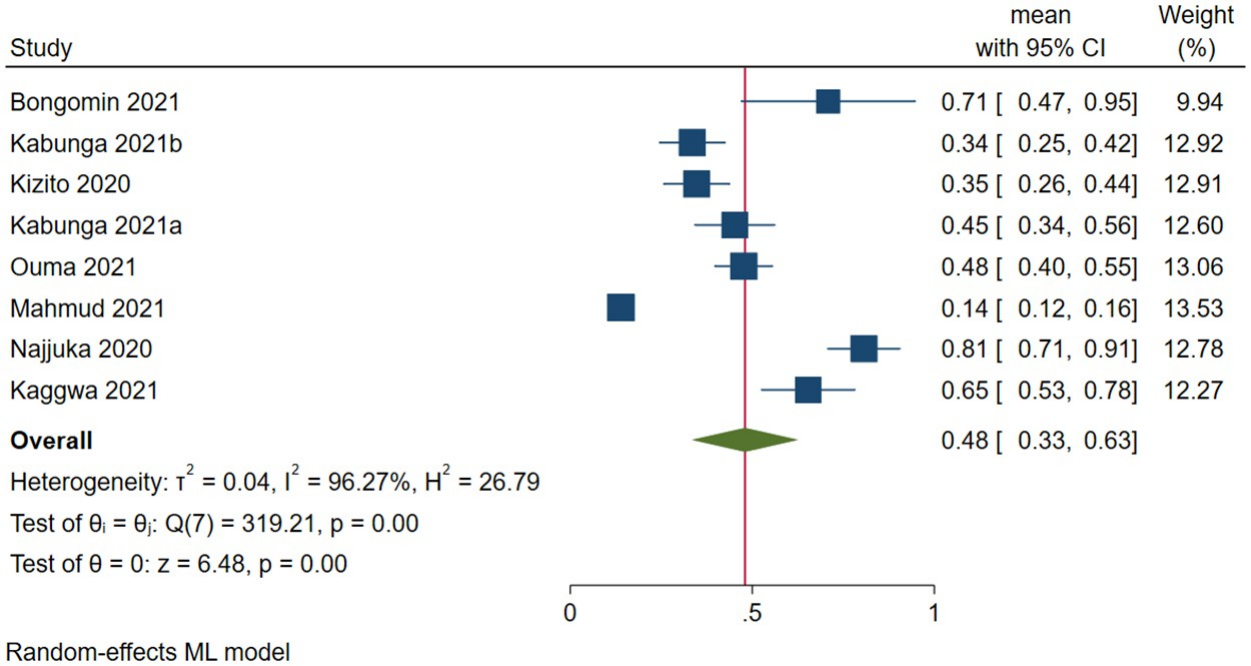
Forest plot on the prevalence of depression during the COVID-19 pandemic.

Due to the high heterogeneity, a subgroup analysis was performed. There were significant differences between the COVID-19 pandemic period (*Q* difference [*QD*] = 5.37, *p*<0.021), the assessment tool used for screening and/or diagnosing depression (*QD* = 2205.32, *p*<0.001), and the type of assessment tool used, i.e., diagnostic (DSM V, DSM VI, MINI, MINI-KID, and clinical diagnosis) or non-diagnostic (*QD* = 8.03, *p* = 0.005) (Table 2). The pooled prevalence of depression during the COVID-19 pandemic was higher than before the pandemic (48.1% vs. 29.3%). Fig 3 shows the forest plot of the prevalence of depression during the COVID-19 pandemic.

**Table 2:** Subgroup analysis of the prevalence of depression in Uganda.

Categories	Subgroups	Number of studies	Pooled prevalence (95% CI)	Q	I^2^	*p*-value	Group difference χ^2^ (p-value)
Pandemic	Pre-pandemic	119	29.3 (25.5–33.0)	23453.03	99.81	<0.001	**5.37 (0.021**)
	During the pandemic	8	48.1 (32.6–63.6)	319.21	96.74	<0.001	
Type of Study	Cross-sectional	92	31.3 (26.7–35.9)	17035.98	99.84	<0.001	1.06 (0.588)
	Cohort	32	29.0 (22.5–35.5)	3616.12	99.43	<0.001	
	Case-control	1	26.7 (19.0–34.5)	0	NA	NA	
Tools used	BDI	3	20.5 (2.9–38.1)	256.48	98.75	<0.001	**2205.32 (<0.001)**
	CASI-5	1	4.9 (3.7–6.1)	0	NA	NA	
	CDI	2	36.6 (5.9–67.3)	83.04	98.80	<0.001	
	CES-D	4	31.1 (11.8–50.4)	225.02	98.60	<0.001	
	Clinician diagnosis	1	0.4 (0.2–0.5)	0	NA	NA	
	DASS-21	1	80.7 (70.9–90.5)	0	NA	NA	
	DHSCL	34	33.7 (27.3–45.4)	2022.12	99.25	<0.001	
	DSM V	1	9.8 (8.3–11.3)	0	NA	NA	
	DSM IV	1	39.8 (31.8–47.7)	0	NA	NA	
	EPDS	4	27.1 (13.4–40.8)	49.28	96.62	<0.001	
	EQ-5D-5L	1	70.8 (47.0–94.6)	0	NA	NA	
	HADS	2	37.3 (15.5–59.0)	12.66	92.10	<0.001	
	KADS-6	1	26.4 (22.3–30.5)	0	NA	NA	
	MINI	24	24.9 (18.1–31.8)	1296.48	99.16	<0.001	
	MINI-KID	6	17.6 (7.7–27.4)	253.96	98.93	<0.001	
	Study designed tool	3	31.5 (-0.9–63.9)	450.27	99.47	<0.001	
	PHQ-2	2	36.1 (-4.77.7)	174.21	99.43	<0.001	
	PHQ-9	31	31.0 (21.9–40.1)	3475.21	99.78	<0.001	
	PROMIS	1	10.6 (10.0–11.2)	0	NA	NA	
	SRQ-20	2	59.4 (29.6–89.1)	13.75	92.72	<0.001	
	SRQ-25	4	33.4 (6.7–60.1)	154.00	98.20	<0.001	
	Self-report	2	41.9 (3.8–80.0)	191.34	99.48	<0.001	
	YSR	1	20.9 (17.1–24.8)	0	NA	NA	
Tool diagnostic	No	94	33.2 (28.6–37.7)	10489.46	99.68	<0.001	**8.03 (0.005)**
	Yes	33	30.5 (26.7–34.2)	3258.96	99.65	<0.001	

DHSCL = Depression sub-section of the Hopkins Symptom Checklist; PHQ = Patient Health Questionnaire (PHQ); MINI = Mini-International Neuropsychiatric Interview; MINI-KID = Mini-International Neuropsychiatric Interview for Children and Adolescents; SRQ = Self-Reporting Questionnaire; CES-D = Center for Epidemiological Studies Depression Scale; EPDS = Edinburgh Postpartum Depression Scale; BDI = Beck Depression Inventory; CDI = Children Depression Inventory; HADS = Hospital Anxiety and Depression Scale; DSM = Diagnostic and Statistical Manual of Mental Disorders; CASI-5 = Child and Adolescent Symptom Inventory-5; DASS-21 = 21-item Depression Anxiety and Stress Scale; EQ-5D-5L = European Quality of Life Five Dimension-Five Level anxiety/depression dimension; KADS-6 = Kutcher Adolescent Depression Scale; PROMIS = Patient-Reported Outcomes Measurement Information System Depression Short Form

### Prevalence of depression in different study populations in Uganda

**Fig 4:**
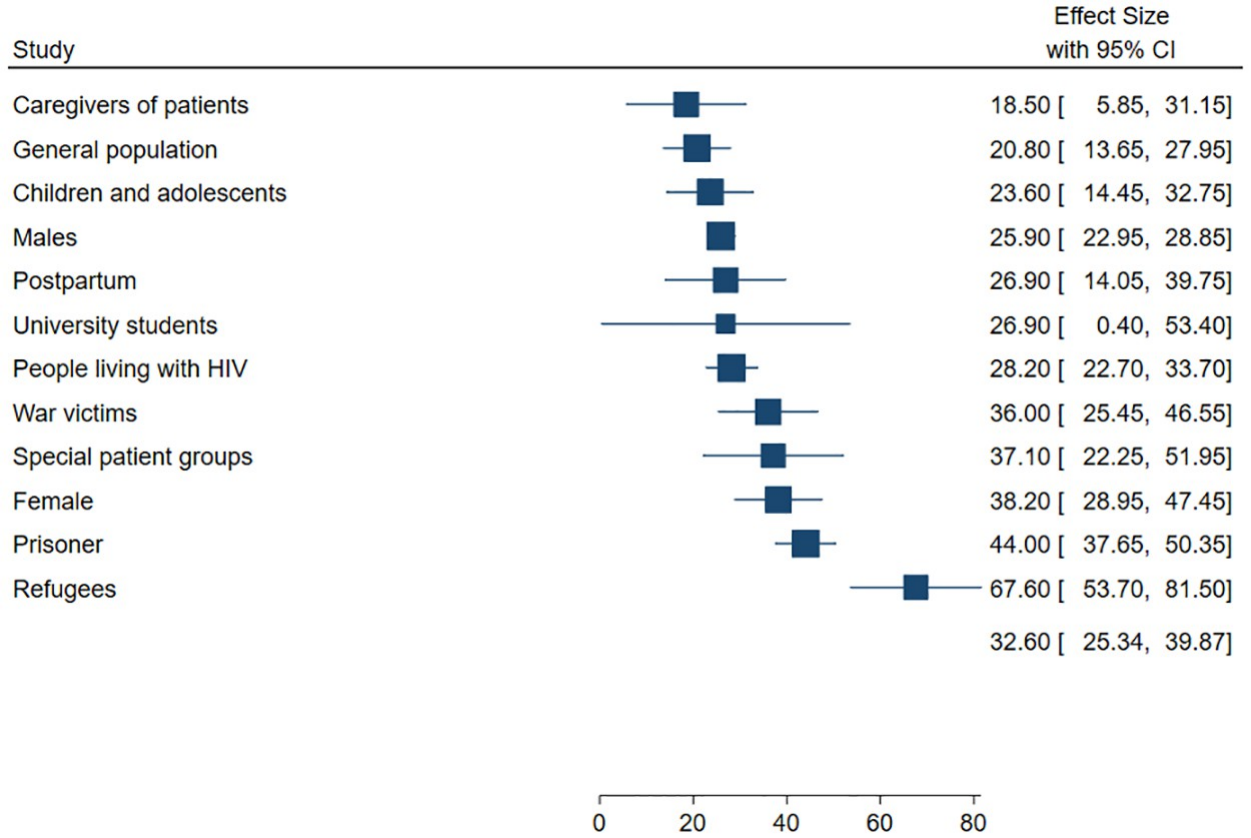
Prevalence of depression in different study groups in Uganda.

Depression was screened or diagnosed in the following study groups in Uganda: (i) individuals living with HIV (*n* = 43) [15, 34, 60, 64, 68, 70–73, 79, 81, 82, 85–91, 93–96, 98–105, 112–114, 117, 119, 123, 127, 128, 130, 132, 133, 136, 141–143, 145, 148–150, 160], (ii) females only (*n* = 25) [16, 31, 35, 52, 59, 73, 79, 97, 113, 116, 134, 139, 144, 158, 166, 170], including seven studies among pregnant or postpartum women [53, 59, 73, 97, 139, 144, 158], (iii) general population (*n* = 19) [55, 61–63, 78, 92, 110, 118, 120, 125, 140, 146, 151, 156, 167, 171], (iv) war victims (*n* = 12) [28, 29, 66, 67, 74–77, 80, 83, 84, 108, 111, 135, 166], (v) special patient groups (such as elderly, outpatients, diabetes mellitus, post-tuberculosis lung diseases, physically ill, post-stroke, cancer, tuberculosis patients, sickle cell disease, patients with stoma, rheumatoid arthritis) (*n* = 12) [17, 21, 31–33, 54, 57, 109, 115, 122, 124, 131, 152], (vi) children and adolescents (*n* = 10) [6, 17, 51, 58, 75–77, 84, 85, 107, 114, 129, 146, 157, 163, 164, 169], (vii) refugees (*n* = 8) [35–37, 116, 153, 159, 162, 168], (viii) caregivers of patients (*n* = 6) [106, 132, 134, 137, 155, 161], (ix) university students (*n* = 4) [14, 56, 65, 165], (x) prisoners (*n* = 1) [154], and males only (*n* = 1) [121] (S1 Table). The pooled prevalence of depression across the different study groups was highest among refugees (67.6%) and lowest among caregivers of patients (18.5%) (Fig 4).

### Depression among refugees in Uganda

**Fig 5:**
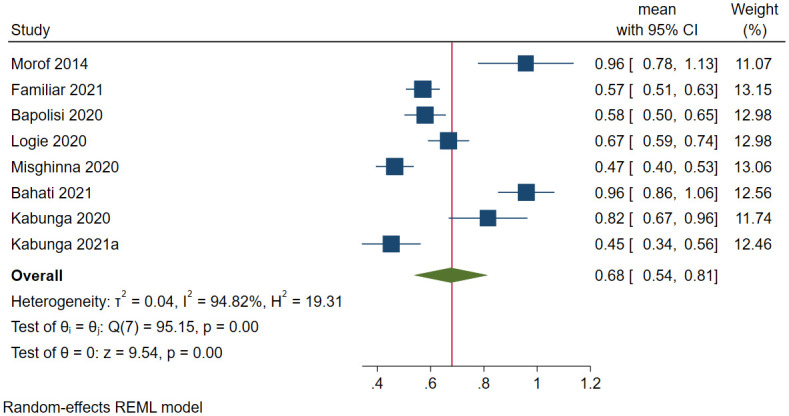
Forest plot on the prevalence of depression among refugees in Uganda.

A total of 2,538 refugees were assessed for depression in Uganda, and 1,652 screened positive for depression in eight studies. The prevalence of depression ranged between 45.2% [168] and 96% [36]. The pooled prevalence of depression was 67.6% (95 CI: 53.7%-81.5%; *I*^*2*^ = 94.82, *p*<0.001) (Fig 5). The estimated slope from Egger’s test was 6.67 (*SE* = 3.037, *p*<0.0281), suggesting publication bias due to small study effects. The funnel plot showed publication bias on visual inspection. A sensitivity analysis was performed using studies within the funnel [37, 159], and the pooled prevalence of depression was 72.7% (95 CI: 58.5%-87.0%; *I*^*2*^ = 67.51, *p*<0.001). Based on meta-regression, the prevalence decreased with the increase in mean age (*β* = -0.07, *SE* = 0.02, *p*<0.001). Other factors were not significant.

#### Depression among studies involving only females in Uganda

**Fig 6:**
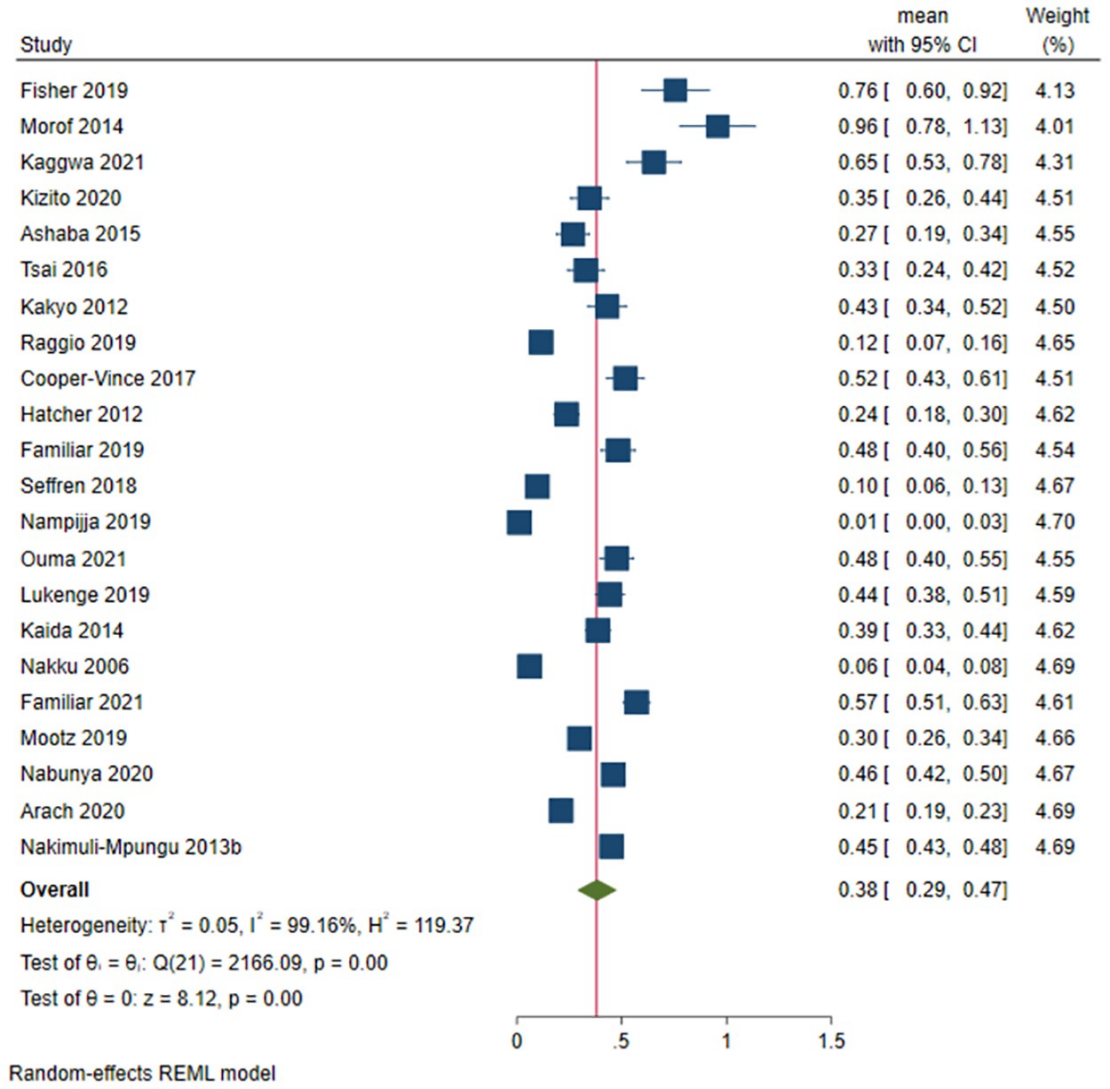
Forest plot on the prevalence of depression among females in Uganda.

A total of 4,222 out of 11476 females had depression in 25 female-only studies. The prevalence of depression ranged between 6.1% [59] and 92.0% [35]. The pooled prevalence of depression was 38.2% (95 CI: 29.0%-47.5%; *I*^*2*^ = 99.16, *p*<0.001) (Fig 6). The funnel plot showed asymmetrical distribution, therefore showing publication bias. The estimated slope from Egger’s test was 8.65 (*SE* = 0.1.428, *p*<0.001), suggesting publication bias due to small study effects. Following meta-regression, no factor significantly affected the prevalence of depression among females in Uganda.

#### Depression among postpartum or pregnant women in Uganda

**Fig 7:**
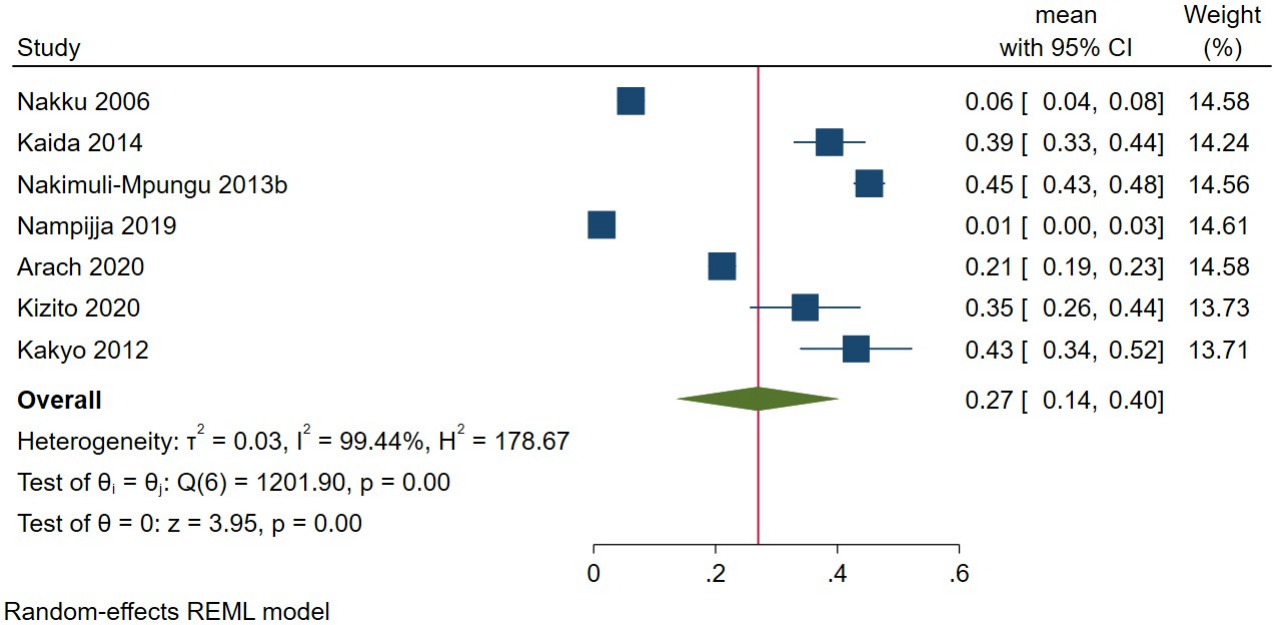
Forest plot on the prevalence of depression among postpartum or pregnant females in Uganda.

Out of the 6296 postpartum or pregnant women in Uganda, 2028 had depression in seven studies. The prevalence of depression ranged between 1.3% [139] and 45.2% [83]. The pooled prevalence of depression among postpartum or pregnant women was 26.9% (95 CI: 13.6%-40.3%; *I*^*2*^ = 99.44, *p*<0.001) (Fig 7). The estimated slope from Egger’s test was 6.76 (*SE* = 3.694, *p*<0.067), suggesting no publication bias due to small study effects. Based on meta-regression, the prevalence of depression among postpartum women statistically significantly decreased with use of MINI (*β* = -0.35, *SE* = 0.11, *p* = 0.002) and increased when the study design was cohort (*β* = 0.22, *SE* = 0.11, *p* = 0.045).

#### Depression among special patient groups in Uganda

**Fig 8:**
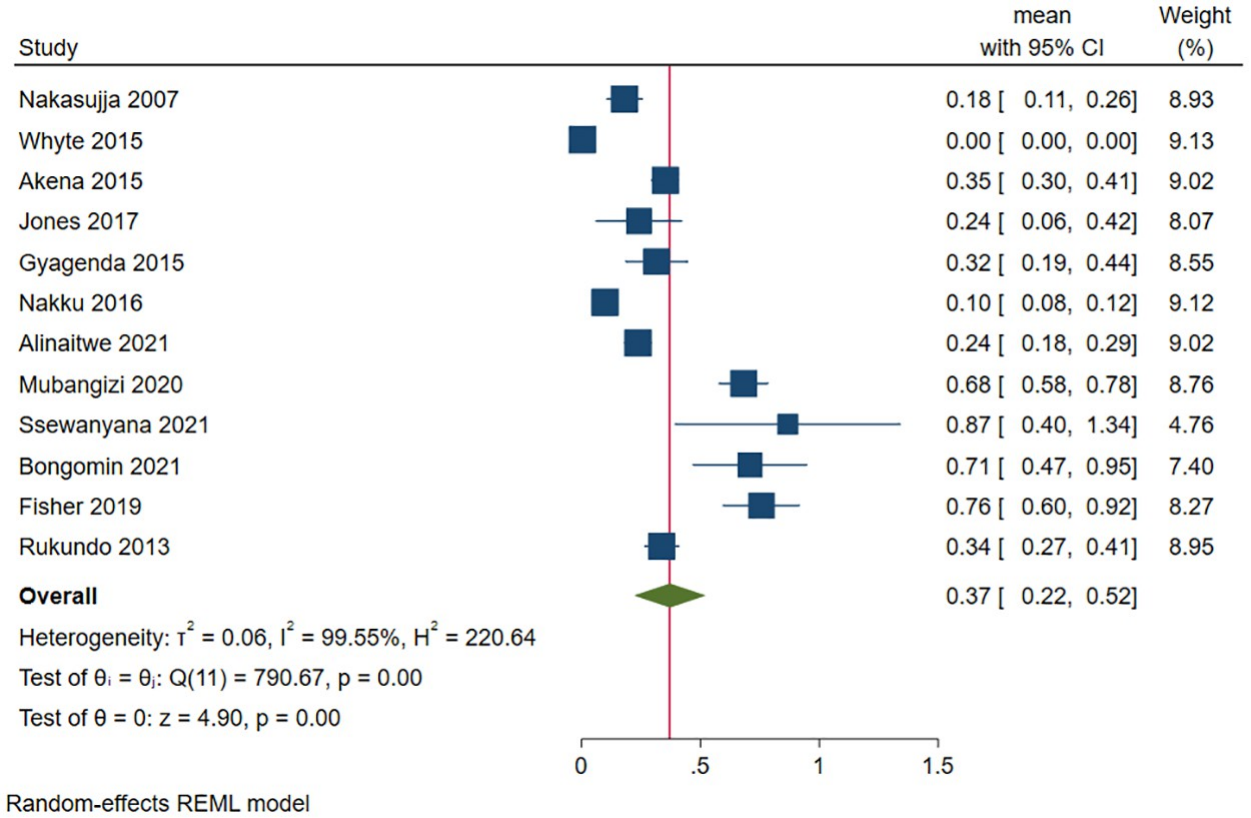
Forest plot on the prevalence of depression among special patient groups in Uganda.

The prevalence of depression ranged from 0.4% among outpatients in northern Uganda [109] and 88% among patients with stomas [32]. The prevalence among those who (i) were elderly was 18% [57], (ii) had tuberculosis was 23.7% [152], (iii) had post-tuberculosis lung diseases was 24% [122], (iv) had cancer was 26% [17], (v) with post-stroke was 31.5% [131], (vi) were physically ill was 33.7% [124], (vii) had diabetes mellitus was 34.8% [115], (viii) had sickle cell disease was 68.2% [54], and (ix) had rheumatoid arthritis was 70.8% [33]. The pooled prevalence of depression from the 14,405 special patient groups (of whom 855 had depression) in 12 studies was 37.1% (95 CI: 22.3%-52.0%; *I*^*2*^ = 99.55, *p*<0.001) (Fig 8). The estimated slope from Egger’s test was 3.82 (*SE* = 1.124, *p*<0.001), suggesting publication bias due to small study effects. Due to the significant heterogeneity, a sensitivity analysis was performed with studies within the funnel, and [115, 122, 124, 131], the pooled prevalence of depression in these studies was 33.8% (95 CI: 29.8%-37.9%; *I*^*2*^ = 0.03, *p*<0.001). At meta-regression, the prevalence of depression among special patient groups statistically significantly increased with increase in the mean age (*β* = 0.01, *SE* = 0.004, *p* = 0.009) and use of SRQ-20 to assess depression (*β* = 0.75, *SE* = 0.30, *p* = 0.013).

#### Depression among war victims in Uganda

**Fig 9:**
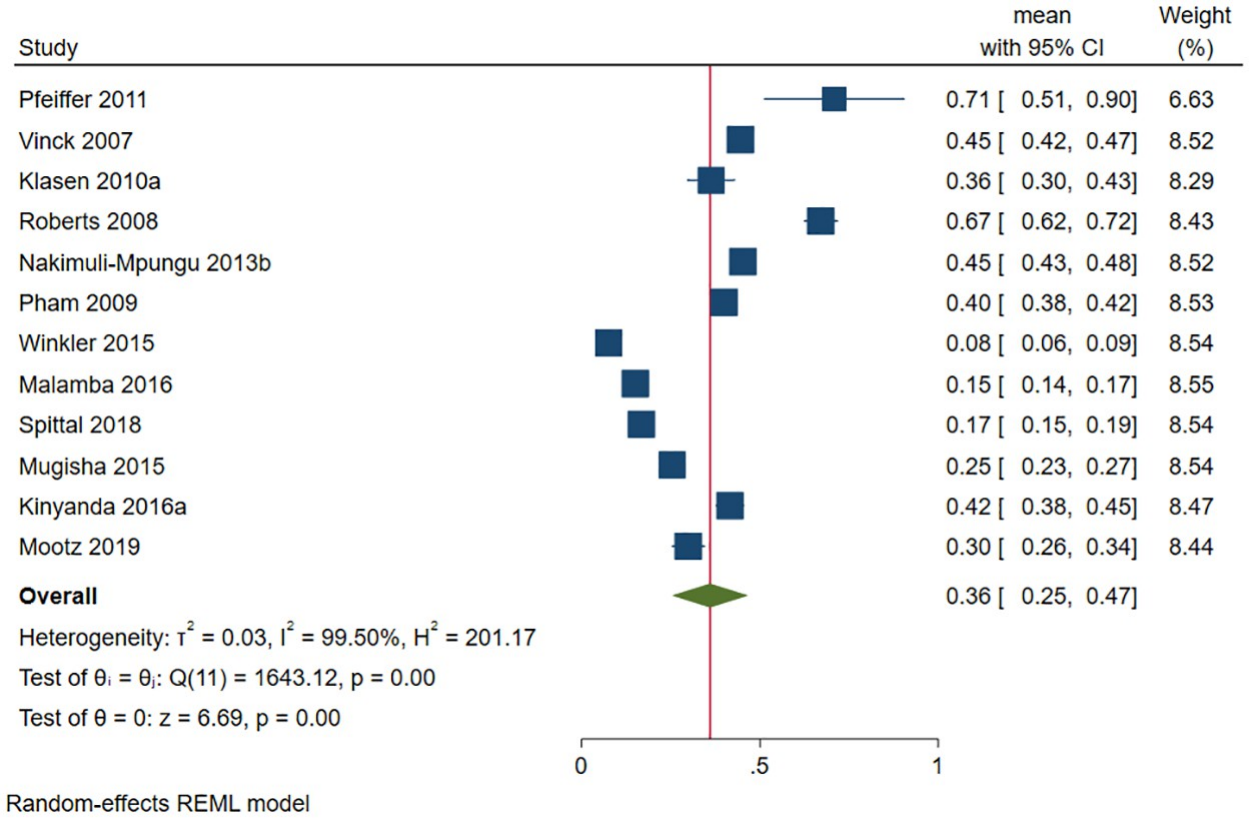
Forest plot on the prevalence of depression among war victims in Uganda.

A total of 6583 (out of 19255) war victims had depression in 12 studies. The prevalence of depression ranged between 7.6% [84] and 71% [67]. The pooled prevalence of depression was 36.0% (95 CI: 25.5%-46.6%; *I*^*2*^ = 99.50, *p*<0.001) (Fig 9). The estimated slope from Egger’s test was 5.24 (*SE* = 2.109, *p* = 0.013), suggesting publication bias due to small study effects. Only one study was within the funnel [75]. At meta-regression, no factor significantly affected the prevalence of depression among war victims in Uganda.

#### Depression among individuals living with HIV in Uganda

**Fig 10:**
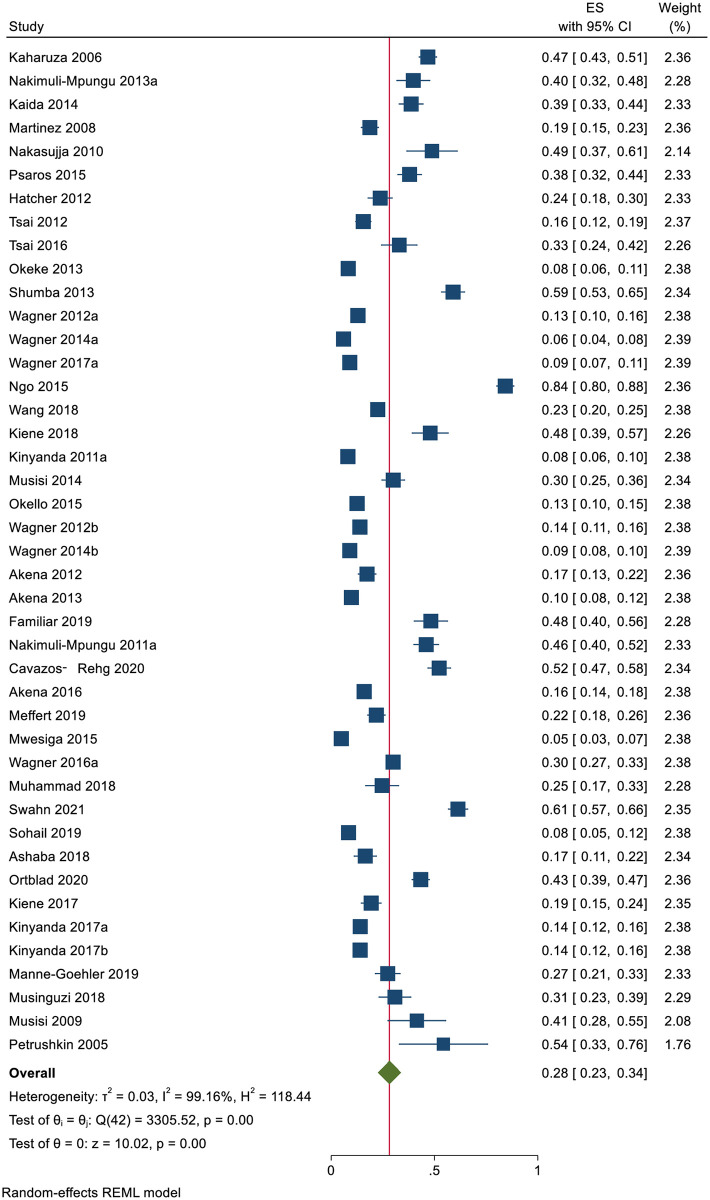
Forest plot on the prevalence of depression among individuals living with HIV in Uganda.

A total of 7704 (out of 26255) individuals living with HIV had depression in 43 studies. The prevalence of depression ranged between 5% [119] and 84% [34]. The pooled prevalence of depression was 28.2% (95 CI: 22.7%-33.7%; *I*^*2*^ = 99.16, *p*<0.001) (Fig 10).

The estimated slope from Egger’s test was 5.72 (*SE* = 0.1.406, *p*<0.001), suggesting publication bias due to small study effects. At meta-regression, no factor statistically significantly affected the prevalence of depression among HIV patients in Uganda.

#### Depression among university students in Uganda

**Fig 11:**
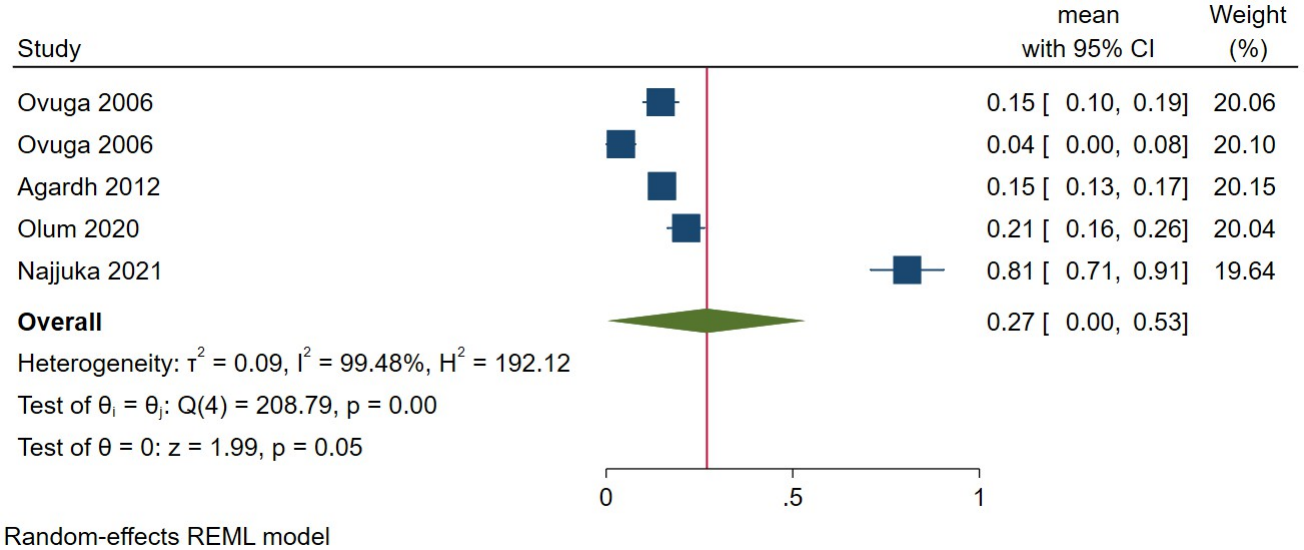
Forest plot on the prevalence of depression among university students in Uganda.

A total of 517 (out of 1982) university students had depression in five studies. The prevalence of depression ranged between 0.4% [56] and 80.7% [14]. The pooled prevalence of depression among university students was 26.9% (95 CI: 0.4%-53.4%; *I*^*2*^ = 99.48, *p*<0.001) (Fig 11). The estimated slope from Egger’s test was 19.85 (*SE* = 4.566, *p*<0.001), suggesting publication bias due to small study effects. The pooled prevalence from the studies within the funnel during sensitivity analysis was 14.9% (95 CI: 12.7%-17.0%; *I*^*2*^ = 0.04, *p*<0.001). The prevalence of depression among students was significantly higher when the DASS-21 was used to screen for depression (*β* = 0.71, *SE* = 0.10, *p*<0.001) and when the study was conducted during the COVID-19 pandemic (*β* = 0.67, *SE* = 0.09, *p*<0.001).

#### Depression among children and adolescents in Uganda

**Fig 12:**
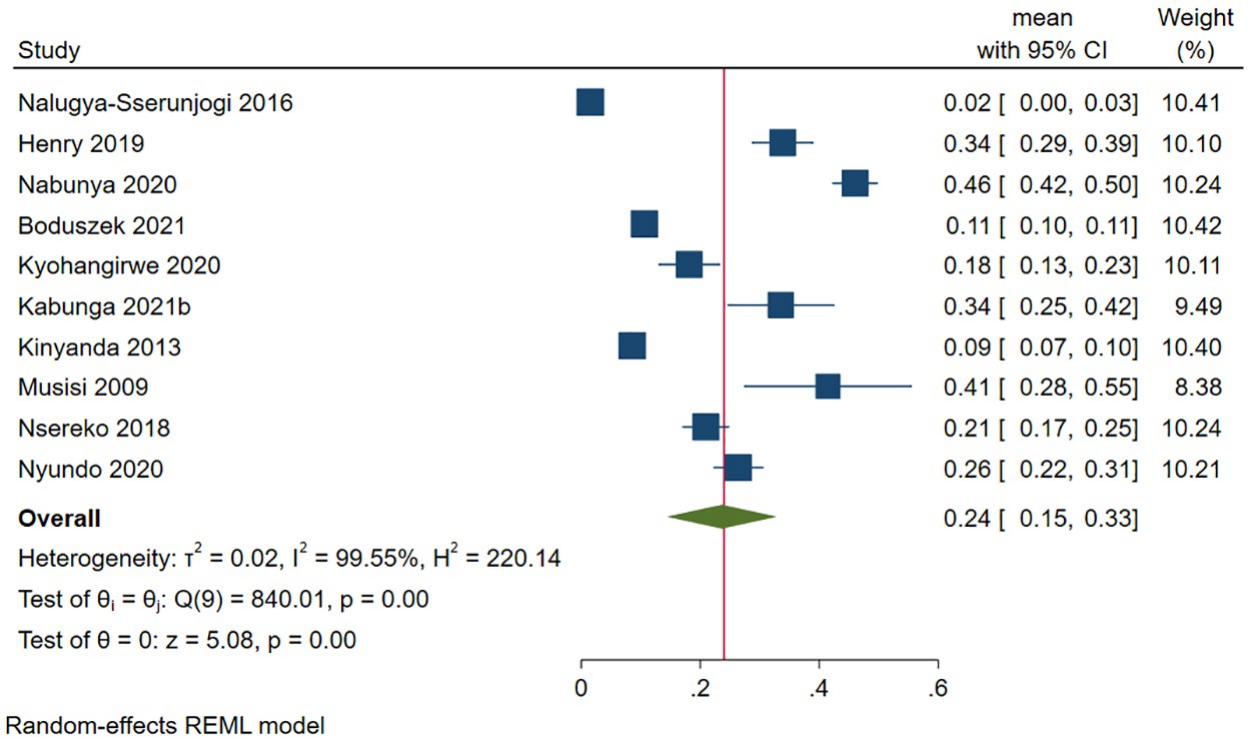
Forest plot on the prevalence of depression among children and adolescents in Uganda.

A total of 2535 (out of 17072) children and adolescents in Uganda screened positive for depression in 10 studies. The prevalence of depression among children and adolescents ranged from 2.9% [58] to 46.03% [157]. The pooled prevalence of depression among children and adolescents was 23.6% (95 CI: 14.5%-32.8%; *I*^*2*^ = 99.55, *p*<0.001) (Fig 12).

The estimated slope from Egger’s test was 5.27 (*SE* = 2.008, *p* = 0.009), suggesting publication bias due to small study effects. Only two studies were within the funnel [6, 51], and sensitivity analysis based on these studies had a pooled prevalence of 23.6% (95 CI: 18.3%-29.0%; *I*^*2*^ = 72.52, *p*<0.001). The prevalence of depression was significantly lower when the following assessment tools were used: (i) MINI-KID (*β* = -0.37, *SE* = 0.09, *p*<0.001), (ii) PROMIS (*β* = -0.35, *SE* = 0.11, *p* = 0.002), and (iii) YSR (*β* = -0.25, *SE* = 0.12, *p* = 0.032).

#### Depression among caregivers of patients in Uganda

**Fig 13:**
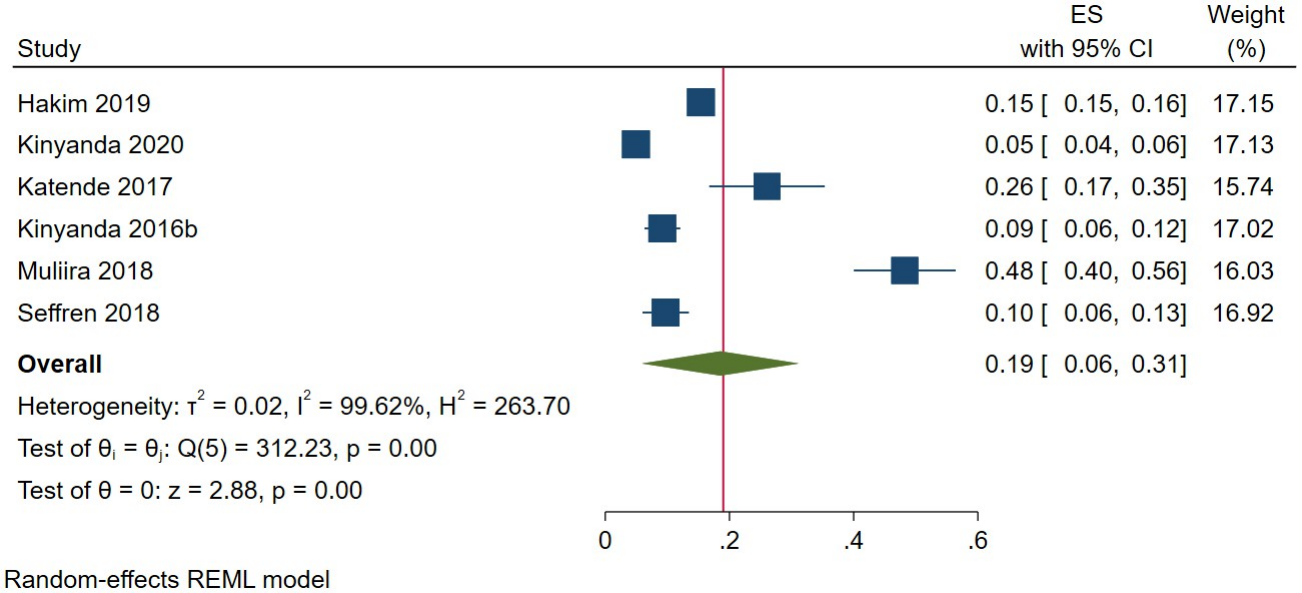
Forest plot on the prevalence of depression among caregivers of selected patient groups in Uganda.

Different types of caregivers were included in this review and they included caregivers for the following patients: individuals living with (i) HIV (*n* = 3) [112, 132, 155], cancer (*n* = 2) [106, 161], and (iii) mental health illness (*n* = 1) [137]. A total of 2189 (out of 14727) caregivers had depression. The pooled prevalence of depression was 18.5% (95 CI: 5.9%-31.2%; *I*^*2*^ = 99.62, *p*<0.001) (Fig 13). The estimated slope from Egger’s test was 6.88 (*SE* = 2.710, *p*<0.011), suggesting publication bias due to small study effects. Only one study was inside the funnel [106]. At meta-regression, no factor statistically significantly affected the prevalence of depression among caregivers of patients in Uganda.

#### Depression among the general population

**Fig 14:**
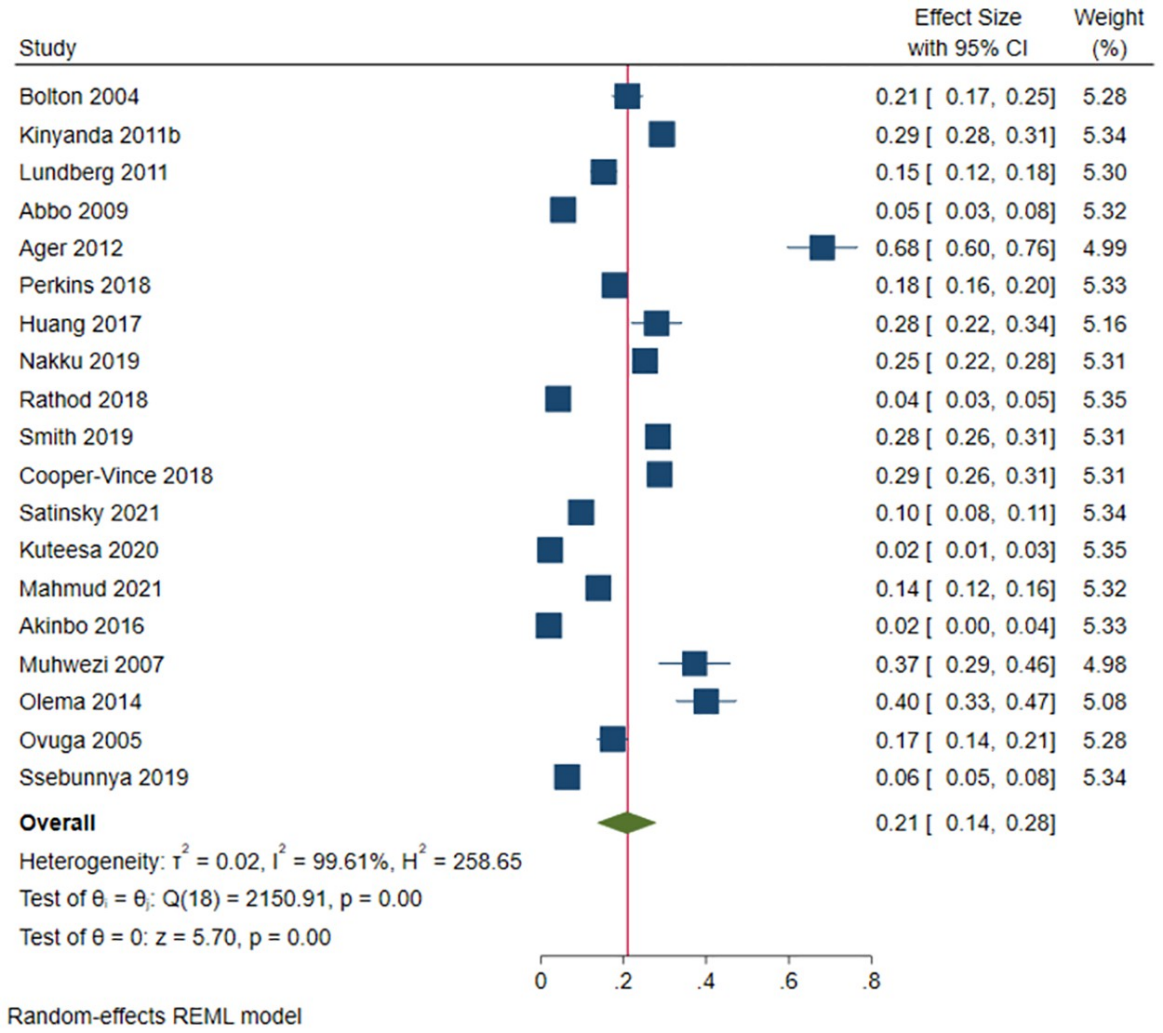
Forest plot on the prevalence of depression among the general population in Uganda.

A total of 4,250 (out of 21,347) members of the general population screened positive for depression in 19 studies. The prevalence of depression ranged between 2.0% among individuals in a fishing community [156] and 68% among national humanitarian aid workers [92]. The pooled prevalence of depression was 20.8% (95 CI: 13.6%-27.9%; *I*^*2*^ = 99.61, *p*<0.001) (Fig 14). The estimated slope from Egger’s test was 10.91 (*SE* = 1.889, *p*<0.001), suggesting publication bias due to small study effects. At meta-regression, no factor statistically significantly affected the prevalence of depression among the general population in Uganda.

## Discussion

The present systematic review and meta-analysis pooling data of close to 124,000 Ugandans collected between 2000 and 2021 showed that approximately one in three individuals had depression. This finding is much higher than the global depression rate of 3.8% [2]. This large difference may be because the majority of the studies included in this review involved study populations that are at higher risk of developing depression, such as refugees, war victims, individuals living with HIV, and caregivers of patients, among others [172–175]. However, the prevalence of depression in Uganda was slightly higher than 27% from a previous systematic review and meta-analysis of the prevalence of depression among outpatients [13].

The prevalence of depression was also higher than previously obtained pooled prevalence rates of depression in Uganda (21.2% among adults and 20.2% among children for studies published between 2010 and 2018 [12]). Since all the previous review studies are included in this study, the difference between the pooled prevalence of depression between the present study and the previous reviews may be due to the effect of the COVID-19 pandemic that led to increased levels of depression [176]. This was clearly indicated by the subgroup analysis, which showed a higher difference between the pre-pandemic pooled prevalence of depression and that during the pandemic. The present systematic review had a different prevalence than the former studies because it included more studies which could have resulted in the pooled prevalence rate being closer to “the true value” of the prevalence of depression in Uganda.

The prevalence of depression in the different study groups was highest among refugees (67.6%) compared to other groups. This prevalence was over twice as high as a previously reported prevalence of depression among refugees and asylum seekers (31.5%) [172]. Uganda, the world’s fourth largest refugee hosting country, has been host to refugees from Congo, South Sudan, Rwanda, Burundi, Somalia, and Ethiopia, among other countries [25]. The high prevalence of depression may be due to refugees leaving their countries to come to a low-income country that is also affected by multiple health, social, and financial struggles, leaving many refugees with depression or worsening their psychological states [172]. The higher prevalence of depression among refugees compared to other studied groups may be because these refugees, on top of their struggles and settling into a new environment, are also affected by the challenges of the country to which other groups are used to.

Uganda has also been affected by civil wars, especially in the northern part of the country. The prevalence of depression among the war victims in Uganda (36.0%) was higher than the 27% global estimate from a systematic review and meta-analysis of war victims [173]. The psychological impact of civil war, refugees, and wars in the neighboring countries on the victims and the workers may be the high prevalence of depression among the national humanitarian workers compared to the rest of the general population.

Despite the declining prevalence of HIV among Ugandans [177], many individuals were affected by the mental and psychological impacts of HIV, such as depression [38, 178]. The prevalence of depression among individuals living with HIV in Uganda (28.2%) in the present study was lower than the global prevalence of 31% [179]. The lower prevalence may be attributed to the efforts made by many researchers to understand and reduce the burden of depression, as evidenced by the high number of studies regarding depression in the present review.

The prevalence was also lower than the previous prevalence of depression among Ugandans with HIV (30.88%) that involved studies published before 2018 [38]. This difference may be attributed to a few studies being included in the previous review (n = 10) [38]. In Uganda, depression among individuals living with HIV has been studied widely and has been assessed as various risk factors such as depression among caregivers of individuals living with HIV [112, 132, 155]. Based on the present review, caregivers of patients have less depression compared to the patients and other study groups. However, they play an integral role in patient care. The prevalence of depression among special groups of caregivers, such as cancer patient caregivers, was higher (42.3%) [180] compared to the pooled prevalence in the present review. This difference may be attributed to the Ugandan culture, where the caregiving role is shared among all family members and creates family support for the affected caregivers, helping prevent depression [161, 181, 182].

Being female was highly represented in the review, with a total of 25 studies being carried out among female-only studies compared to only one male-only study. This possibly shows neglect of male gender mental health by researchers. Future research should include more studies among males, so that true estimates of the burden of depression can be determined and evidence-based interventions can be designed. Depression among children and adolescents has also been studied more than studies of male adults. The prevalence of depression among children and adolescents (23.6%) was higher than 20.2% among children in Uganda for papers published between 2010 and 2018 [12].

Despite having no missing studies imputed into the overall prevalence, the heterogeneity was high. Following sensitivity analysis, the prevalence of depression in Uganda was 0.9%—a prevalence lower than the estimated global prevalence of 3.8% [2]. Based on the various analyses, the main sources of heterogeneity were (i) the COVID-19 pandemic, where the prevalence of depression was significantly higher than in the period before the pandemic as reported by various researchers and meta-analyses [176, 183]; and (ii) the tools used in screening/diagnosing depression with the DASS-21 detecting significantly higher prevalence rates of depression compared to other study tools. The significant difference may be due to the tool being used during the early stages of the COVID-19 pandemic [14] when many of the individuals were experiencing severe depression due to various stressors [176, 183]. The difference in the reported prevalence of depression could be due to various studies using different assessment tools with different psychometric properties regarding depression. Also, some tools were diagnostic, such as the DSM criteria, while others were screening tools, such as the DASS-21.

### Limitations and recommendations

When interpreting these results, the following limitations need to be considered. First, despite only 16% of the 127 papers not having a total score of nine on the JBI Checklist and the use of random effect models, there were significantly high levels of heterogeneity due to the depression assessment tools and the period of study. Future researchers should conduct reviews of studies with fewer variations, especially in relation to the tools used to assess depression. However, for better quality and to increase reliability in future meta-analyses, future researchers should continue using the commonly used tools such as PHQ-9, DHSCL, and MINI. Also, the classification of the different study groups in the present study may have caused heterogeneity in the included studies, for example, among the general population. Second, some of the included studies were prone to recall biases since all their data were based on self-report. Third, despite data from various regions and districts in Uganda being presented, a large majority of the country was still not represented. This suggests more research regarding depression in other parts of this multicultural and multilingual country should be conducted and/or a nationally representative survey study [18]. Moreover, despite conducting a detailed literature search, some of the common databases (e.g., *EMBASE*, *CINAHL*) and journals that publish papers on mental health illness were not included. Therefore, some studies could have been missed. Also, the search strategy did not include some of the common terms associated with depression, such as mental health, psychological disorder/problem, and mood. It is recommended that future studies include sources for unpublished data to generalize the findings better.

While the meta-analysis was comprehensive and provided a broader picture of the prevalence of depression in various populations, it is still difficult to generalize the results because the prevalence of depression in Uganda in many regions was not represented, and different populations’ generalizations or groupings were subjective (e.g., humanitarian workers). Future studies within these populations and across wider regions in the country would be helpful in implementing treatments according to targeted needs (socioeconomic, cultural, refugee-status, etc.).

## Conclusion

In the present meta-analysis, the synthesized data showed that approximately one in three individuals in Uganda has depression, which was highest among refugees and other special populations. Interventions for active screening, diagnosis, and management of depression among the general population and special populations and cohorts are recommended.

## Supporting information

S1 Fig Funnel plot for the included studies about depression in Uganda. (TIF)

S2 Fig (TIF)

S1 Table Prevalence of depression in study populations in Uganda. (DOCX)
