# Supplementary figures and images for: Prevalence of depression in Uganda: A systematic review and meta-analysis
Source: PLoS One. 2022 Oct 20;17(10):e0276552. doi: 10.1371/journal.pone.0276552 (PMC9584512; doi:10.1371/journal.pone.0276552)

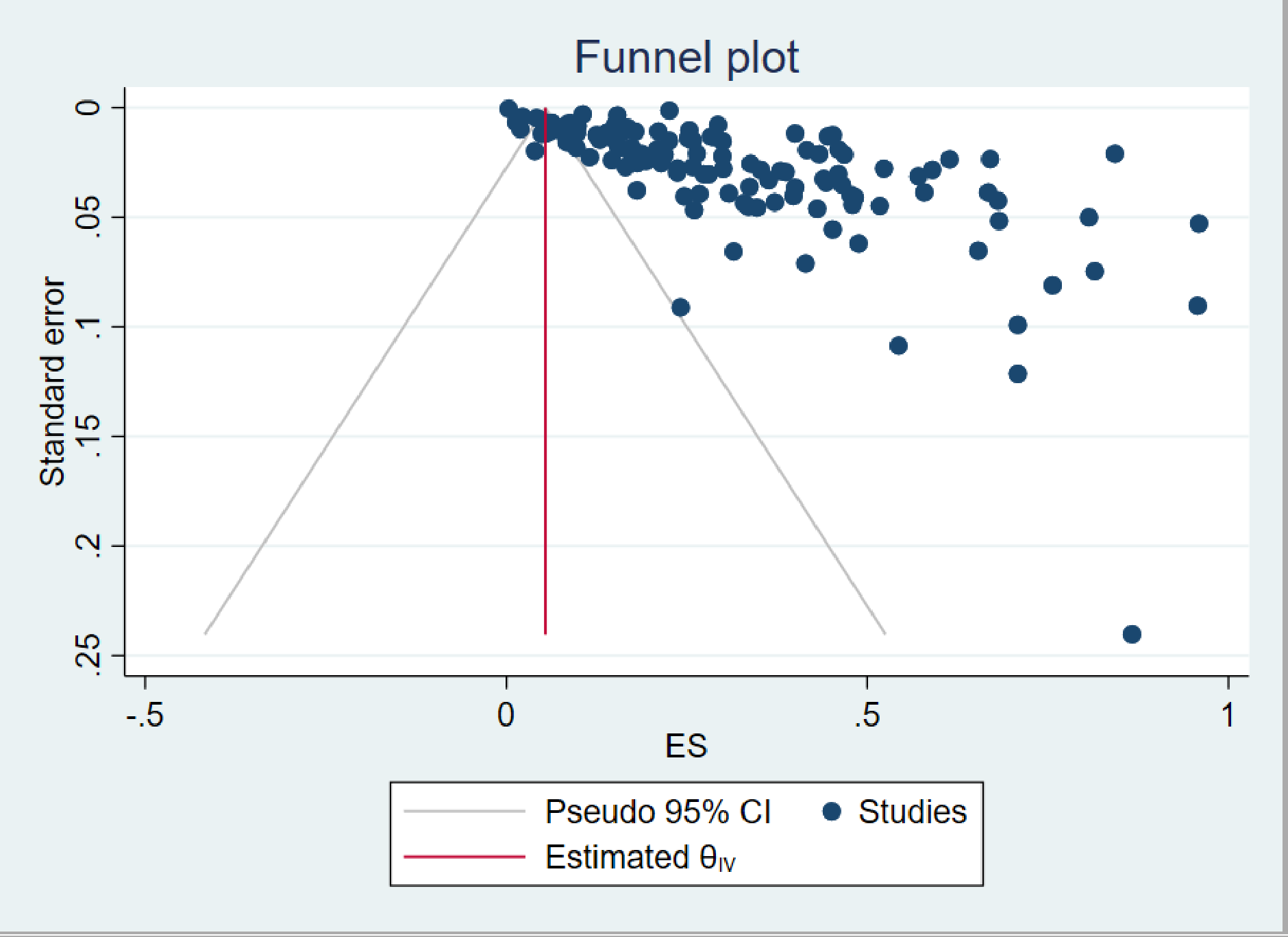

Supplement: S1 Fig — (TIF) [file pone.0276552.s001.tif]

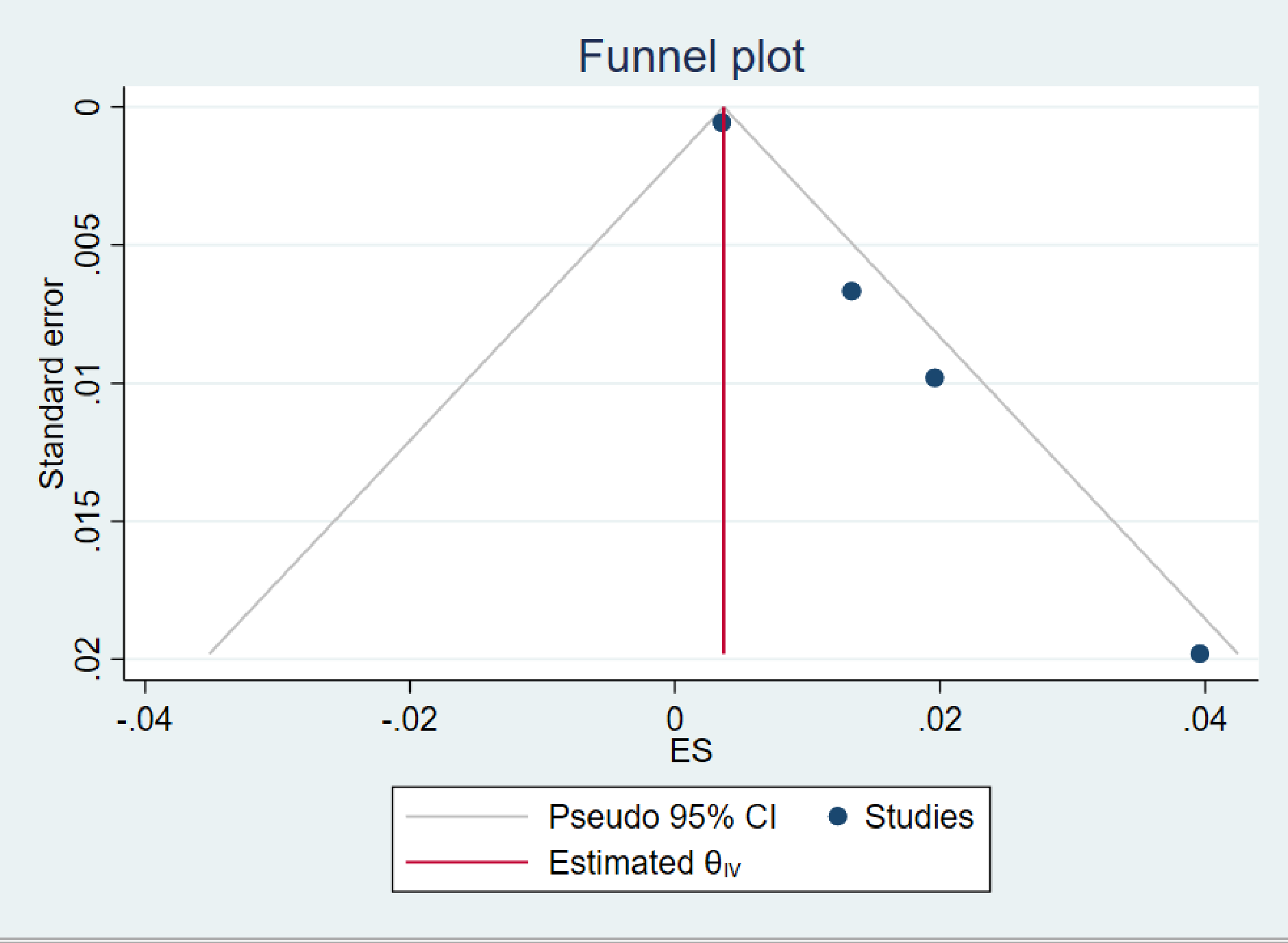

Supplement: S2 Fig — (TIF) [file pone.0276552.s002.tif]
