# Supplementary material for: Prevalence of depression in Uganda: A systematic review and meta-analysis
Source: PLoS One. 2022 Oct 20;17(10):e0276552. doi: 10.1371/journal.pone.0276552 (PMC9584512; doi:10.1371/journal.pone.0276552)
Supplement: S1 Table — (DOCX) [file pone.0276552.s003.docx]

**S1 Table: Prevalence of depression in study populations in Uganda**

| Group | Number of studies | Pooled prevalence (95% CI) | Small sample effect -eggers | Q | I^2^ | *P* value |
| --- | --- | --- | --- | --- | --- | --- |
| Female | 25 | 38.2 (29.0-47.5) | Yes | 2166.09 | 99.16 | <0.001 |
| Male | 1 | 25.9 (22.9-28.8) | NA | NA | NA | NA |
| University students | 4 | 26.9 (0.4-53.4) | Yes | 208.79 | 99.48 | <0.001 |
| Pregnant or Postpartum women | 7 | 26.9 (14.6-40.3) | Yes | 1201.90 | 99.44 | <0.001 |
| Special patient groups | 12 | 37.1 (22.3-52.0) | Yes | 790.67 | 99.55 | <0.001 |
| Children and adolescents | 10 | 23.6 (14.5-32.8) | Yes | 840.01 | 99.55 | <0.001 |
| Caregivers of selected patients | 6 | 18.5 (5.9-31.2) | Yes | 312.23 | 99.62 | <0.001 |
| Refugees | 8 | 67.6 (53.7-81.5) | Yes | 95.15 | 94.82 | <0.001 |
| War victims | 12 | 36.0 (25.5-46.6) | Yes | 1643.12 | 99.50 | <0.001 |
| Individuals living with HIV | 43 | 28.2 (22.7-33.7) | Yes | 3305.52 | 99.16 | <0.001 |
| General population | 19 | 20.8 (13.6-27.9) | Yes | 2150.91 | 99.61 | <0.001 |
| Prisoners | 1 | 44.0 (37.6-50.3) | NA | NA | NA | NA |
