# Supplementary material for: Prevalence of depression in Uganda: A systematic review and meta-analysis
Source: PLoS One. 2022 Oct 20;17(10):e0276552. doi: 10.1371/journal.pone.0276552 (PMC9584512; doi:10.1371/journal.pone.0276552)
Supplement: Attachment — Submitted filename: Review report.docx [file pone.0276552.s004.docx]

**Review report**

**Comments to the author**

I would like to thank the editor for giving me the chance to act as an academic reviewer of this interesting work from Uganda. I am really impressed by this paper starting from its title to the way it is presented. I will have few issues as I have pointed below to be considered by the authors.

1. The authors should use the PRISM-P instead of the PRISMA guideline for the systematic review and meta-analysis.
2. The authors reported as they excluded those observational studies which were included in previous systematic review studies in the PRISMA flow diagram. This is against your justification of this study which is to determine the comprehensive pooled prevalence of depression in Uganda. I would like to the author to strongly consider this and explain how the inclusion of these studies will have a problem in their study result.
3. The authors reported as they have conducted sensitivity analysis since there was heterogeneity between studies. What sources of heterogeneity were identified as a result of your sensitivity analysis? Explain and better to include in the manuscript
4. The authors added too many figures in this paper; I would like to advise them to minimize the number of tables and figures.
5. Similarly, the authors used too many references. Better to limit by avoiding the outdated references used.
6. The discussion section is interesting but it is satisfactory as of using 173 references. Your study can best be supported by more scientific evidences in addition to your presented evidences. I strongly advice to make it stronger.
